# From Bench to Breath: Material Integrity and Performance of Filtering Facepiece Respirators and Surgical Masks After Multi-Cycle Dry-Heat Reprocessing

**DOI:** 10.3390/microorganisms14010069

**Published:** 2025-12-29

**Authors:** Mohammad Sagor Hosen, José G. B. Derraik, Mohammad Shahbaz, William A. Anderson, Yvonne C. Anderson, Mark P. Staiger

**Affiliations:** 1 Department of Mechanical Engineering, University of Canterbury, Christchurch 8140, New Zealand; hosensagor@gmail.com (M.S.H.); mark.staiger@canterbury.ac.nz (M.P.S.); 2 Department of Paediatrics: Child and Youth Health, Faculty of Medical and Health Sciences, University of Auckland, Auckland 1023, New Zealand; yvonne.anderson@curtin.edu.au; 3 Environmental-Occupational Health Sciences and Non-Communicable Diseases Research Centre, Research Institute for Health Sciences, Chiang Mai University, Chiang Mai 50200, Thailand; 4 Liggins Institute, University of Auckland, Auckland 1023, New Zealand; mohammad.shahbaz@auckland.ac.nz; 5 Department of Chemical Engineering, University of Waterloo, Waterloo, ON N2L 3G1, Canada; wanderso@uwaterloo.ca; 6 Curtin Medical School, Faculty of Health Sciences, Curtin University, Bentley, WA 6102, Australia; 7 The Kids Research Institute Australia, Perth, WA 6009, Australia; 8 Child and Adolescent Community Health, Child and Adolescent Health Service, Perth, WA 6000, Australia

**Keywords:** airflow, breathability, decontamination, disinfection, FFR, particle filtration efficiency, healthcare workers, N95, pandemic preparedness, personal protective equipment, PPE

## Abstract

Dry heat inactivates pathogens on personal protective equipment without chemical residues, but its effects on material integrity and performance across multiple reprocessing cycles have not been comprehensively assessed. We evaluated five filtering facepiece respirator (FFR) models and three surgical mask (SM) models after one, two, and three cycles of dry heat (80 °C, 90 min). We measured fabric and strap tensile properties as indicators of mechanical durability [Young’s modulus (*E*), yield strength (*σ_y_*), ultimate tensile strength (*σ_UTS_*), and strain at failure (*ε_f_*)]. We also assessed particle filtration efficiency (PFE) and airflow resistance (breathability). Under the methods applied herein, all untreated SMs and FFRs performed within the range anticipated for their type. Tensile properties exhibited heterogeneous, model-specific responses to thermal stress. FFR fabrics ranged from progressive stiffening (Dräger DR-X1720C; +120% *E*) to marked softening (3M-8210; −82% *E*), while SM fabrics exhibited softening, consistent with thermal relaxation. Straps made of thermoplastic elastomer (3M-8210 and 3M-9320A+) weakened (15–31% *σ_UTS_* decrease), whereas braided polyisoprene straps (3M-1860S and 3M-1870+) maintained their original strength. Despite these changes, all treated FFR replicates met filtration requirements across all cycles (45/45). For SMs, 24/27 treated replicates met the required PFE threshold (≥98%), but 3 treated RH-S919B replicates fell below this threshold (PFE 94.9% and 97.7% after one cycle, and PFE 97.3% after three cycles), identifying a potential model-specific vulnerability to the treatment. Breathability remained within control ranges for most models; however, the Level 2 ZA-S001B showed decreased breathability (higher airflow resistance) after two (+11.1 Pa) and three (+13.3 Pa) dry-heat cycles, whereas the Level 3 RH-S920TFG showed modest improvements in breathability (lower airflow resistance, up to −10.1 Pa). Under these laboratory conditions, up to three cycles of dry heat at 80 °C for 90 min preserved PFE and breathability in all treated FFR replicates and in most treated SM replicates. Nonetheless, there were measurable, component-specific mechanical changes (especially in some straps) that could compromise fit and durability with repeated use. These findings support dry heat at 80 °C for 90 min as a potential component of emergency PPE processing strategies, provided that model-specific quantitative fit testing and extended-wear studies confirm safe real-world reuse, regulatory approvals are met, and end-user acceptability is considered.

## 1. Introduction

The COVID-19 pandemic, caused by the novel coronavirus SARS-CoV-2, generated a global public health crisis that markedly increased the demand for personal protective equipment (PPE) [1]. For healthcare professionals and other frontline workers at elevated risk of SARS-CoV-2 infection via respiratory droplets and aerosols, surgical masks (SMs) and filtering facepiece respirators (FFRs) became indispensable tools for protection.

This surge in demand, estimated by the World Health Organization (WHO) to involve a 40% increase in face mask production, quickly overwhelmed global supply chains, resulting in widespread shortages that compromised the safety of frontline workers [1,2]. PPE shortages were common in wealthy countries such as New Zealand (NZ) [3], the USA [4] and European countries [5], but were more severe in low- and middle-income countries (LMICs) [6,7,8,9]. During the COVID-19 pandemic, healthcare facilities in LMICs could not meet basic levels of staff and patient protection [6,7]; however, this problem was widespread even before the pandemic started [8].

Notably, this public health challenge also resulted in a burgeoning environmental crisis. FFRs and SMs are predominantly manufactured from petroleum-based polymers (primarily polypropylene) and are designed for single use [10]. The sharp increase in PPE production and subsequent disposal has resulted in a substantial influx of plastic waste into terrestrial and aquatic ecosystems, where it can persist for decades, eventually breaking down into harmful microplastics that also pose a threat to wildlife and environmental health [11,12,13,14,15]. Discarded PPE has become an important contributor to the global plastic waste burden [11,12,13,14,15], and this issue was recognised in a WHO report in 2022 [16]. Pandemic PPE management must address supply resilience and sustainability, including recycling damaged PPE and validating the treatment of intact PPE for safe reuse where required to reduce environmental impact [10,12,17,18].

During the pandemic PPE crisis, various treatment methods were investigated to enable their safe reuse, with “decontamination systems” (i.e., bioburden reduction) for FFRs (N95 masks) authorised by the U.S. Food and Drug Administration (FDA) [4,19]. While the immediate pressures of the pandemic have subsided worldwide—with authorities such as the FDA revoking emergency use authorisations for PPE disinfection systems [19,20]—the need for these strategies remains critical. Epidemiological modelling suggests a high probability of novel pandemic emergence in the coming decades [21]. Once again, the world will most likely face increased PPE demand, greater disruptions to global transportation, disproportionate PPE distribution, as well as the need to minimise environmental harm. In this scenario, widespread PPE shortages in LMICs (and even in high-income countries) are likely to recur, particularly during periods of economic stress. Establishing robust and accessible disinfection methods is crucial to enable the safe reuse of PPE and build supply chain resilience [1,2].

Disinfection processes must not compromise the function and safety of treated PPE (particularly SMs and FFRs), as they must continue to offer the required protection to users (particularly healthcare workers) and patients, and meet regulatory approvals. These techniques primarily include chemical processing, ultraviolet germicidal irradiation (UVGI), and heat treatment [22,23,24,25,26,27]. Chemical methods using agents such as bleach (sodium hypochlorite) or alcohol can effectively inactivate pathogens, including SARS viruses [23,28,29,30]. However, alcohol degrades the electrostatic charge of the polypropylene filter layers, which are critical for efficient particle capture, thereby compromising their protective function [24,31]. While bleach may not have the same impact on the electrostatic charge, it impacts FFR materials in other ways [24,32] and also leaves a residual odour toxic for the wearer [32,33].

UVGI is effective against pathogens without compromising filtration efficiency [27,34,35,36]. However, UVGI efficacy depends on the dose delivered, and it can be impaired by line-of-sight issues, where shadows cast by the complex topography of a mask (e.g., folds, seams) can shield pathogens from irradiation [37]. Further, at least one study reported that UVGI led to a marked reduction in the strength of respirator materials, including visual tears, with the materials easily coming apart when handled [38].

Heat treatment, particularly dry heat, has emerged as a promising alternative due to its efficacy, practicality, low cost, and ease of implementation in diverse settings [27,39,40,41,42,43,44]. Dry heat inactivates viruses by denaturing their structural proteins [45], and it can be effective against SARS-CoV-2 and other pathogens without the drawbacks of chemical residues or shadowing effects [27,46]. However, heat treatment at elevated temperatures may not only affect the polymeric structure, but also the material’s fibre orientation, distribution, and entanglement, altering their properties and impairing the function of SMs and FFRs [27,31,40,47]. Nonetheless, we [37] and others [27,48] have shown that SARS-CoV-2 and other coronaviruses can be inactivated at temperatures as low as 55–70 °C.

Several studies have assessed the function and fit of FFRs and SMs after multiple cycles of dry-heat treatment [27,42,43]. However, few studies have comprehensively examined the effects of dry heat on the underlying material properties of both PPE types. FFRs and SMs are complex, multi-component systems, whose protective function depends not only on the filtration media but also on the structural integrity of all components, including the straps that ensure a tight facial seal. The degradation of these materials is a complex process, and changes in their mechanical properties—such as stiffness, strength, and ductility—are the direct precursors to functional failure. It is therefore essential to determine how repeated dry-heat treatment affects these mechanical properties and whether such changes compromise PPE function.

This study aimed to fill that knowledge gap by systematically examining the effects of multiple cycles of dry-heat treatment on a range of commercially available SMs and FFRs used in NZ. We sought to investigate the potential impacts of up to three cycles of dry-heat treatment on the mechanical properties of SM and FFR fabrics and straps. We also evaluated how these changes may impact critical performance metrics (i.e., particle filtration efficiency and breathability). Providing a mechanistic link between material science and functional safety would yield robust evidence to guide future research and policies on PPE reuse for future pandemic preparedness and environmental sustainability.

## 2. Materials and Methods

### 2.1. PPE Studied

The PPE models were selected from those approved for use by the NZ Ministry of Health, which were widely used during the COVID-19 pandemic. Manufacturer and model names are provided solely for accurate reporting and reproducibility; no comparative ranking, endorsement, or disparagement is intended. Our objective was not to compare brands, but to determine whether a defined dry-heat reprocessing protocol would preserve key laboratory performance metrics relevant to potential reuse for commonly available models in NZ.

The models tested included five FFRs and three SMs (two ASTM Level 2, one Level 3). The specific models, manufacturers, and primary material compositions are detailed in Table 1. All FFR and SM bodies were primarily composed of polypropylene, with one FFR model (3M-8210) also containing polyester. The FFR panel comprised NIOSH-certified N95 respirators (3M 1860S, 3M 1870+, and 3M 8210) and P2/FFP2 class particulate respirators (Dräger DR X1720C and 3M 9320A+); throughout this article, we refer to these collectively as FFRs. Their straps consisted of braided polyisoprene, thermoplastic elastomer, and woven elastic textile materials (Table 1).

For SMs, the ASTM level refers to their ASTM F2100-19 performance classification [49]. Level 2 and Level 3 SMs require the same filtration rates and flammability class, distinguished only by the higher fluid-resistance requirement for Level 3 [50].

### 2.2. Dry-Heat Treatment

The dry-heat disinfection protocol was designed to balance pathogen inactivation efficacy with preserving material integrity and function. Previously, we showed that dry-heat treatment at 60 °C for 45 min, 65 °C for 30 min, or 70 °C for 15 min inactivated SARS-CoV-2 on FFR coupons, resulting in a 7.0-log_10_ TCID_50_/mL reduction [37]. While the work demonstrated that lower temperatures can inactivate SARS-CoV-2 [37], a more rigorous protocol is required to neutralise heat-tolerant bacterial pathogens commonly encountered in healthcare settings [46]. We previously reported that dry heat at 75 °C for 90 min achieved at least a 6-log_10_ reduction in several multidrug-resistant bacteria [37,46]. To provide a safety margin for temperature fluctuations commonly found in standard laboratory or domestic ovens, a target temperature of 80 °C was selected for this study.

Treatments were conducted in a domestic fan-forced oven with a 3000 W heating element (model OB60SC7CEPX1, Fisher & Paykel Appliances Ltd., Auckland, NZ). No water or humidification was added to the chamber, and the protocol was intended as a dry-heat treatment. Relative humidity inside the oven was not measured or controlled. The oven’s temperature stability was assessed before the experiments using nine Type-T thermocouples with an accuracy of ±0.5 °C distributed throughout the chamber. Two 120 min tests were run with temperature measurements every 2 s. After a 60 min stabilisation period, the oven maintained a mean temperature of 79.8 ± 0.5 °C (Figure S1), with all locations remaining within 2.5 °C of the 80 °C target (Figure S2).

PPE materials underwent four treatment regimens: untreated (control), one cycle (1×), two cycles (2×), and three cycles (3×) of dry-heat treatment. For each cycle, individual SMs and FFRs were placed on metal racks inside the pre-heated oven for 90 min. After treatment, items were removed and allowed to cool to ambient room temperature (≈25 °C). For items undergoing multiple cycles, a 24 h period at ambient temperature was observed between successive treatments.

### 2.3. Tensile Properties of PPE

The mechanical properties of the PPE materials were evaluated using a uniaxial testing load frame (MTS Criterion C43, MTS Corp., Eden Prairie, MN, USA) equipped with a 500 N load cell [51]. For each PPE model and treatment level, one full-thickness coupon (25 mm × 100 mm) was cut from the outer surface of five replicates using a guillotine (Premium Guillotine Trimmer, Dahle 564, Dahle North America Inc., Peterborough, NH, USA). Coupons were carefully cut parallel to the longest dimension of the mask/respirator, avoiding welds and folds, to preserve the multi-layered structure of the original item.

**Figure 1:**
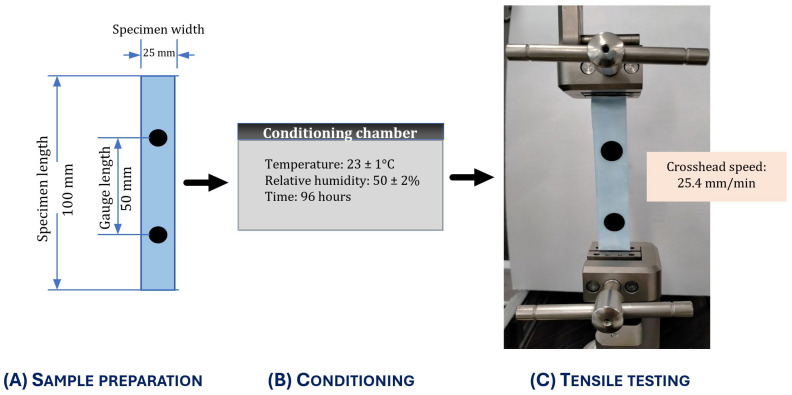
Diagrammatic overview of the tensile testing protocol. (**A**) Specimens (testing sample length 100 mm; width 25 mm for fabric coupons; gauge length 50 mm between marker dots) were prepared for testing. (**B**) Specimens were conditioned at 23 ± 1 °C and 50 ± 2% relative humidity for 96 h. (**C**) Tensile testing was performed with the specimen clamped between grips at a crosshead speed of 25.4 mm/min.

All specimens were conditioned for 96 h at 23 ± 1 °C and 50 ± 2% relative humidity before testing. Tensile tests were performed at a constant crosshead speed of 25.4 mm/min (Figure 1). An FVX02 non-contact video extensometer (MTS Systems, Eden Prairie, MN, USA) equipped with a 50 mm camera lens (Tamron 004375, Tamron Co., Ltd., Saitama-shi, Saitama, Japan) was used to measure strain over a 50 mm gauge length (Figure 1). Rubber-faced grips were used to hold the specimens to minimise damage to the sample layers during testing.

**Figure 2:**
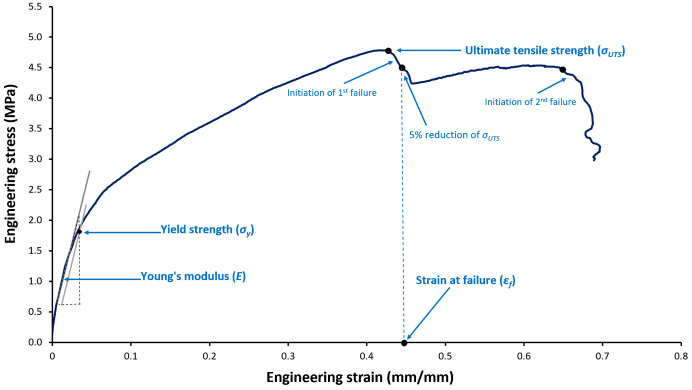
Schematic representation of a stress–strain curve illustrating the four tensile parameters measured in this study. Young’s modulus (*E*) represents the slope of the initial linear elastic region that quantifies material stiffness—represented by the dark gray line overlaying the curve (with the dashed triangle illustrating the slope calculation). Yield strength (*σ_y_*) is the stress at which permanent (plastic) deformation begins, determined using the 0.2% strain offset method—the light gray line represents this offset line drawn parallel to the elastic region, with its intersection with the stress–strain curve defining *σ_y_*. Ultimate tensile strength (*σ_UTS_*) is the maximum stress the material can withstand—corresponding to the curve’s peak. Strain at failure (*ε_f_*) is the strain at the onset of failure, defined here as the point on the post-peak curve where measured stress first falls by 5% from *σ_UTS_* (i.e., to 0.95 × *σ_UTS_*) after reaching *σ_UTS_*, serving as an operational marker of failure initiation (partial loss of load-bearing capacity)—this point is indicated by the vertical blue dashed line. Strain is presented here in dimensionless units (mm/mm), but it may also be expressed as a percentage (mm/mm × 100).

The testing methodology was adapted from the ASTM D828-16 standard [51]. Although standards specific to non-woven textiles exist (e.g., ASTM D5035-11 [52]), ASTM D828-16 was selected because its procedural framework is particularly well suited to the thin, paper-like structure of the mask materials and provides a rigorous, repeatable basis for analysis. The following parameters were calculated from the resulting tensile stress–strain curves (adapted from ISO 527-1 [53]), also illustrated in Figure 2:

- Young’s (tensile) modulus (*E*)—A measure of tensile stiffness (quantifying its resistance to elastic deformation) calculated as the ratio of stress to strain within the initial linear elastic region.
- Yield strength (*σ_y_*)—The stress at which the material begins to deform plastically (permanently) as determined from the 0.2% strain offset method.
- Ultimate tensile strength (*σ_UTS_*)—The maximum tensile stress that the material can withstand before failure.
- Strain at failure (*ε_f_*)—Defined here as the engineering strain at which the measured engineering stress on the post-peak curve first falls to (or below) 0.95 × *σ_UTS_* (i.e., a 5% drop from *σ_UTS_*) after *σ_UTS_* is reached. This criterion was selected as a reproducible operational marker of failure initiation (at least partial loss of load-bearing capacity) rather than complete rupture, consistent with prior tensile work [54]. Strain was calculated from video extensometer–measured surface displacement using a 50 mm gauge length.

The interpretation of potential treatment effects on these mechanical properties is summarised in Table 2.

Tensile load (*F*) was recorded by the testing machine; engineering stress was calculated as per Equation (1).

(1) $$\sigma =F÷\;A_{0}$$

where *A*_0_ is the initial cross-sectional area (width × thickness). Stress–strain curves were subsequently constructed from *F* and video-extensometer displacement (50 mm gauge length).

### 2.4. Particle Filtration Efficiency and Airflow Resistance

Particle filtration efficiency (PFE) and airflow resistance (breathability—Δ*P*; Pa) were measured for three replicates of each SM and FFR model at each treatment level using a Palas PMFT-1000 instrument (Palas GmbH, Karlsruhe, Germany) at Nanolayr Ltd. (Auckland, NZ). Testing protocols were aligned with the relevant regulatory standards for each PPE type. Since fit was not assessed, PFE data should be interpreted independently of respirator seal performance. Accordingly, any reuse implementation must include fit testing (quantitative or qualitative) compliant with OSHA 29 CFR 1910.134 [55] to ensure an adequate seal in practice.

FFRs were tested according to the National Institute for Occupational Safety and Health (NIOSH) TEB-APR-STP-0059 standard [56]. Complete respirators were challenged with a charge-neutralised polydisperse sodium chloride (NaCl) aerosol at a flow rate of 85 L/min. The test aerosol had a count median diameter (CMD) of 0.075 ± 0.02 µm (geometric standard deviation < 1.86), which is near the most penetrating particle size (MPPS) typically around ≈50 nm (30–60 nm range) for N95-class electret filter media [56]. Each tested filter must have a PFE ≥95% when challenged with the specified NaCl aerosol [56].

SMs were tested according to the ASTM F2299/F2299M-03(2017) standard [57], with their performance assessed against the ASTM F2100-19 classification, which requires PFE ≥ 98% for both Level 2 and Level 3 SMs [49]. Material coupons (central panel, avoiding seams) were challenged with non-neutralised 0.1 µm polystyrene latex particles at a flow rate of 28.3 L/min [57].

PFE was determined by measuring the ratio between the downstream and upstream particle concentrations (Equation (2)).

(2) $$PFE\left(\%\right)=\left(1-\frac{C_{\;downstream}}{C_{\;upstream}}\right)\times 100$$

While the breathability (Δ*P*) was measured for all samples, the values are instrument-specific (Palas PMFT-1000 test area/flow) and were not compared with regulatory thresholds. These laboratory procedures are not certification tests, and results should not be interpreted as manufacturer specifications or regulatory compliance.

### 2.5. Blood Penetration Resistance of Surgical Masks

Exploratory synthetic-blood splash testing of SMs is described in Appendix A. These tests should not be interpreted as a standard-compliant assessment, but rather as a means to provide information on potential changes in properties resulting from treatment cycles.

### 2.6. Statistical Analyses

Descriptive statistics (mean and standard deviation) were calculated for all quantitative outcomes. After confirming the assumptions of normality and homogeneity of variance, tensile property data were compared between treatment groups using a one-way analysis of variance (ANOVA). Planned pairwise comparisons between treated and control samples were assessed using Fisher’s Least Significant Difference (LSD) tests.

Particle filtration efficiency and airflow resistance data did not meet parametric assumptions, and the non-parametric Kruskal–Wallis test was used to assess overall group differences. Due to small sample sizes for each treatment level (*n* = 3), individual pairwise *p*-values were constrained by discrete distribution limitations; thus, pairwise effect magnitudes were quantified using Wilcoxon-derived Hodges–Lehmann location shift estimates with 95% confidence intervals (CI) if there was evidence of a treatment effect (i.e., CI did not cross zero).

The number of replicates according to PPE type and test is summarised in Table S1. Statistical analyses were performed using SAS v9.4 (SAS Institute, Cary, NC, USA) and SPSS v29.0 (IBM Corp, Armonk, NY, USA). All tests were two-sided, and a *p*-value < 0.05 was considered statistically significant. Given the safety-critical nature of PPE integrity, our statistical approach prioritised sensitivity to material degradation or loss of function (minimising Type II errors) over the stringent control of family-wise error rates (Type I errors). The risk of failing to identify a compromise in protective function (a false negative) far outweighs the risk of erroneously flagging a safe batch as degraded (a false positive), i.e., adjustment for multiplicity can be counterproductive for safety-critical assessments [58]. Thus, no statistical correction was applied to the planned pairwise comparisons to the control samples. This approach ensures a conservative safety assessment, treating any statistically detectable mechanical alteration as a potential risk signal requiring further investigation.

## 3. Results

### 3.1. Scope and Baseline Performance

Under the laboratory methods used here, all untreated (control) PPE performed within the range anticipated for their type. All products tested are designated for single use, and any heat treatment constitutes off-label use. Consequently, post-treatment performance should not be assumed to meet regulatory or manufacturer specifications, and any changes observed under these off-label conditions must not be interpreted as defects for the products’ intended single-use purpose.

### 3.2. Mechanical Properties of Surgical Mask Fabrics

Tensile testing of surgical mask fabrics revealed distinct response patterns in mechanical properties (Table 3 and Table S2).

The ZA-S001B (L2) exhibited softening (loss of stiffness) characteristics (Table S2). Young’s modulus decreased after one (−10.0 MPa; *p* < 0.0001) and two cycles (−12.4 MPa; *p* < 0.0001) compared with controls, but unexpectedly returned to near-baseline values after three cycles (*p* = 0.81) (Table 3). Yield strength remained unchanged after one cycle (*p* = 0.63) but decreased after two (−0.21 MPa; *p* = 0.015) and three cycles (−0.84 MPa; *p* < 0.0001). *σ_UTS_* progressively decreased after one (−0.88 MPa; *p* = 0.0004), two (−0.71 MPa; *p* = 0.003), and three cycles (−1.11 MPa; *p* < 0.0001) (Table 3). Strain at failure showed non-linear changes, decreasing after one (−11.3%; *p* = 0.006) and three cycles (−10.2%; *p* = 0.012) but not after two cycles (*p* = 0.49) (Table 3).

The Level 2 RH-S919B displayed an unusual annealing-like response (Table S2). While Young’s modulus consistently decreased after one (−24.1 MPa), two (−17.3 MPa), and three cycles (−31.0 MPa) compared to controls (all *p* < 0.0001), both yield strength (*σ_y_*) and *σ_UTS_* increased after multiple cycles (Table 3). Specifically, *σ_y_* increased after two (+0.78 MPa; *p* = 0.001) and three cycles (+1.51 MPa; *p* < 0.0001) compared to controls (Table 3). *σ_UTS_* decreased initially after one cycle (−0.60 MPa; *p* = 0.005) but then increased substantially after two (+1.73 MPa; *p* < 0.0001) and three cycles (+1.49 MPa; *p* < 0.0001) compared to controls (Table 3). Most notably, strain at failure more than doubled after two (+21.3%; *p* = 0.0006) and three cycles (+19.3%; *p* = 0.002) compared to controls (Table 3), indicating that repeated dry-heat cycles rendered the RH-S919B fabric softer, stronger, and more ductile rather than brittle.

The only Level 3 SM tested (RH-S920TFG) showed consistent softening (loss of stiffness) across all parameters (Table S2). Young’s modulus decreased uniformly from 57.4 MPa (control) by 28–29 MPa after all treatment levels (all *p* < 0.0001) (Table 3). Paradoxically, yield strength (*σ_y_*) increased after all treatments (0.61–0.89 MPa; all *p* < 0.0001), while *σ_UTS_* progressively decreased after one, two (both −0.76 MPa; *p* = 0.0003), and three cycles (−1.39 MPa; *p* < 0.0001) (Table 3). The strain at failure decreased substantially after all treatments (from 40.5% to 19.1–27.6%; all *p* < 0.001), consistent with increased brittleness (Table 3).

### 3.3. Blood Penetration Resistance Outcomes (Surgical Masks)

The results of the exploratory synthetic-blood splash challenge are provided in Appendix A and are not standard-compliant; they should not be used to infer post-treatment fluid-barrier compliance. Nonetheless, only 1 out of 45 tests of treated SMs showed any apparent performance degradation under the conditions used.

### 3.4. Mechanical Properties of FFR Fabrics

The filtering facepiece respirator (FFR) fabrics exhibited highly variable and model-specific responses to thermal stress (Table 4 and Table S2). There were heterogeneous changes in fabric stiffness. The Dräger DR-X1720C fabric showed progressive stiffening consistent with possible embrittlement (Table S2), with Young’s modulus increasing from a mean of 5.19 MPa in the control by +4.3 MPa after one cycle (*p* < 0.0001), +3.6 MPa after two cycles (*p* = 0.0003), and +6.2 MPa after three cycles (*p* < 0.0001) (Table 4).

By contrast, three of four 3M models tested displayed reduced stiffness after two or three treatments. The most substantial softening occurred in the 3M-8210 model, with Young’s modulus decreasing by 82% after a single cycle (−52.4 MPa; *p* < 0.0001) (Table 4), indicative of severe loss of stiffness (large modulus reduction) consistent with stress-relaxation and/or morphology change, which was also observed after two (−36.8 MPa; *p* < 0.0001) and three cycles (−46.3 MPa; *p* < 0.0001). In contrast, the results for 3M-9320A+ were contradictory, with *E* increasing after one cycle (*p* < 0.0001) but subsequently decreasing after two or three cycles (Table 4).

For 3M-1860S and 3M-9320A+, *σ_UTS_* and ductility (*ε_f_*) were unaffected at all treatment levels (Table 4). Among 3M-8210 fabric samples, *σ_UTS_* was reduced after two (−1.21 MPa; *p* = 0.0001) or three (−0.92 MPa; *p* = 0.002) cycles, with *ε_f_* unaffected throughout (*p* = 0.25; Table 4). There was evidence that *σ_UTS_* in the 3M-1870+ was reduced after one (−0.92 MPa; *p* = 0.002) and two cycles (−0.49 MPa; *p* = 0.067) but not after three treatments (*p* = 0.18) (Table 4). There were conflicting findings on *σ_UTS_* for Dräger DR-X1720C fabrics, which was lower after one cycle (−0.45 MPa; *p* = 0.023) but increased after two (+0.69 MPa; *p* = 0.001) and three (+0.46 MPa; *p* = 0.021) cycles, while *ε_f_* was reduced by ≈15 percentage points after one or three cycles (both *p* < 0.001) and by ≈7 percentage points after two cycles (*p* = 0.059) (Table 4). Lastly, changes in yield strength (*σ_y_*) were inconsistent across models, with no clear, unifying pattern of mechanical weakening or strengthening (Table 4).

### 3.5. Mechanical Properties of FFR Straps

The elastomeric straps—thermoplastic elastomers (e.g., 3M-8210 and 3M-9320A+) versus thermoset/braided polyisoprene (e.g., 3M-1860S and 3M-1870+)—showed distinct, material-specific responses. Thermoplastic straps tended to lose strength and/or exhibit greater ductility after heat cycles, whereas braided polyisoprene straps showed no detectable changes in *σ_UTS_* across treatments (Table 5).

The straps of the 3M-8210 (thermoplastic elastomer) showed reductions in both *σ_UTS_* (overall *p* = 0.0001) and *σ_y_* (overall *p* < 0.0001) after a single dry-heat treatment cycle (*p* = 0.034 and *p* < 0.0001, respectively), with these effects persisting after multiple treatments (Table 5). For *σ_y_*, mean values fell from 0.43 MPa in controls to 0.08 MPa after one cycle (≈81% reduction; *p* < 0.0001), remaining well below baseline after two (0.16 MPa; *p* = 0.0002) and three (0.19 MPa; *p* = 0.0005) cycles. At the same time, the 3M-8210 straps became more ductile, with *ε_f_* increasing after multiple treatments, specifically after two (*p* = 0.003) or three cycles (*p* = 0.002) (Table 5). Similar but inconsistent results were seen for ductility (*ε_f_*) in 3M-9320A+ straps (thermoplastic elastomer) (Table 5). Regarding *σ_y_*, it increased after two or three cycles in 3M-9320A+ (both *p* < 0.0001) and DR-X1720C (woven elastic textile, latex-free) straps (*p* = 0.003 and *p* = 0.022, respectively) (Table 5).

In contrast to the 3M-8210 and 3M-9320A+ models, the straps of 3M-1860S (braided polyisoprene), 3M-1870+ (braided polyisoprene), and DR-X1720C models displayed no changes in *σ_UTS_* across all treatments (Table 5). Further, Young’s modulus (*E*) decreased after a single treatment in 3M-9320A+ (*p* = 0.016) and 3M-1870+ (*p* < 0.0001) and after two cycles in 3M-1860S (persisting thereafter). Conversely, *E* increased in 3M-8210 after two (*p* = 0.0003) and three (*p* = 0.001) cycles (Table 5). For DR-X1720C, the overall change in *E* was not statistically significant (*p* = 0.064), although the two-cycle vs. baseline pairwise comparison was significant (*p* = 0.009) (Table 5).

**Figure 3:**
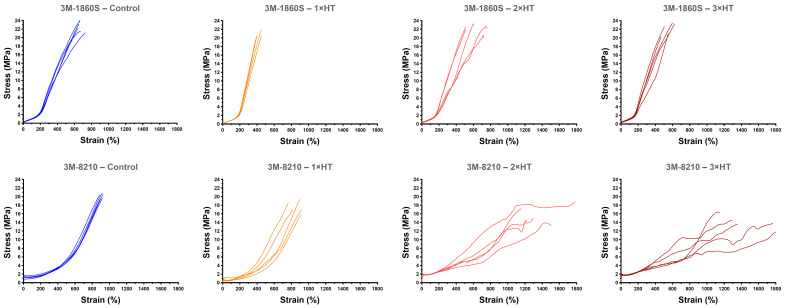
Stress–strain curves (replicate-level) for filtering facepiece respirator (FFR) straps composed of braided polyisoprene (3M-1860S) and thermoplastic elastomer (3M-8210) across dry-heat treatment levels. Each dry-heat treatment cycle consisted of 90 min at 80 °C in a fan-forced oven. Control indicates untreated samples, while 1× HT, 2× HT, and 3× HT indicate one, two, or three sequential treatment cycles. Engineering strain was calculated from video-extensometer displacement over a 50 mm gauge length. Each panel shows all tested replicates for the given model and treatment level (*n* = 5). Traces terminate at the last valid point prior to specimen rupture or complete loss of load; post-rupture segments are not shown. Some 3M-8210 traces exhibit abrupt load drops or non-monotonic behaviour at high strain, consistent with late-stage damage progression and/or intermittent grip effects; quantitative tensile parameters derived from these curves are reported in Table 5. Model names are provided solely for accurate reporting and reproducibility; no comparative ranking, endorsement, or disparagement is intended.

The contrasting mechanical responses of braided polyisoprene and thermoplastic elastomer straps are illustrated in Figure 3, which displays stress–strain curves for the tested replicates of the 3M-1860S and 3M-8210 models across all treatment levels. The 3M-1860S straps (braided polyisoprene) displayed consistent stress–strain behaviour throughout, with tightly clustered curves maintaining peak stresses of 20–24 MPa and strains at failure of 400–750% across all treatment conditions. In contrast, the 3M-8210 straps (thermoplastic elastomer) showed treatment-associated changes with increasing heat cycles, consistent with reduced strength and altered failure patterns: untreated samples reached peak stresses of 19–21 MPa and strains at failure within a very narrow range of ≈890–930%, whereas samples subjected to two or three heat cycles showed reduced peak stresses across a wider range (≈12–19 MPa) and substantially increased strains at failure (≈1150–1800%). The 3M-8210 curves also displayed greater inter-replicate variability after heat treatment compared with controls, suggesting less predictable mechanical behaviour. These stress–strain profiles provide an illustrative visual summary of the divergent material responses quantified in Table 5.

### 3.6. Particle Filtration Efficiency

**Figure 4:**
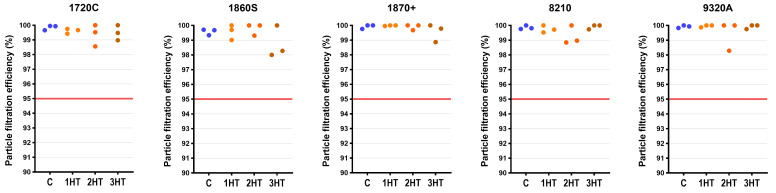
Effects of multiple dry-heat treatment cycles on the particle filtration efficiency of filtering facepiece respirators (FFRs). Each dry-heat treatment cycle consisted of 90 min at 80 °C in a fan-forced oven; C indicates the untreated controls, while 1HT, 2HT, and 3HT indicate one, two, or three sequential cycles under these same conditions. Data are all treated replicates for a given model and treatment level; individual values may be offset (rather than aligned straight) to prevent overlap between data points with identical results. The horizontal red line at 95% indicates the minimum required threshold for FFRs. Model names are provided solely for accurate reporting and reproducibility; no comparative ranking, endorsement, or disparagement is intended. Blue circles indicate untreated control replicates, while heat-treated replicates are shown in shades of orange to red.

Irrespective of any changes in the mechanical properties of SM and FFR materials tested, their primary functional performance in filtration and breathability remained largely stable. Critically, all treated FFR replicates (45/45) maintained PFE above NIOSH N95 filtration requirements (Figure 4; Table S3).

**Figure 5:**
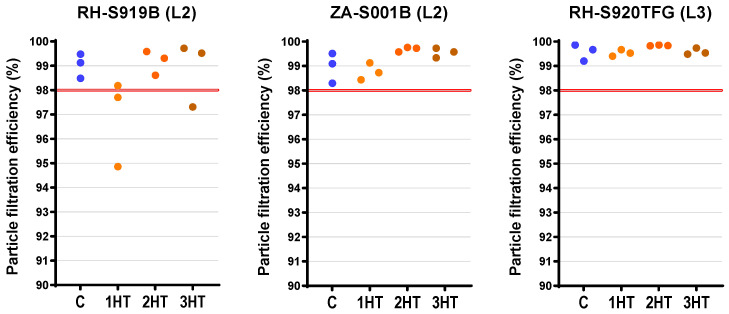
Effects of multiple dry-heat treatment cycles on the particle filtration efficiency of surgical masks. Each dry-heat treatment cycle consisted of 90 min at 80 °C in a fan-forced oven; C indicates the untreated controls, while 1HT, 2HT, and 3HT indicate one, two, or three sequential cycles under these same conditions. Data are all treated replicates for a given model and treatment level; individual values may be offset (rather than aligned straight) to prevent overlap between data points with identical results. Levels 2 (L2) and 3 (L3) refer to their ASTM F2100-19 performance classification, which requires a particle filtration efficiency ≥ 98% [49], with this threshold represented by the horizontal red line. Model names are provided solely for accurate reporting and reproducibility; no comparative ranking, endorsement, or disparagement is intended. Blue circles indicate untreated control replicates, while heat-treated replicates are shown in shades of orange to red.

However, for SMs, two treated RH-S919B replicates failed after one cycle (94.9% and 97.7%) and one replicate failed after three cycles (97.3%) (Figure 5), indicating a model-specific vulnerability in this Level 2 mask under the tested conditions. These failures showed no consistent cycle-related pattern (there were no PFE failures after two cycles), which is compatible with stochastic sample variability or lot-to-lot heterogeneity rather than a monotonic cumulative heat-damage effect. All other treated SM replicates passed the ≥98% ASTM F2100-19 criterion (overall 24/27). While ZA-S001B showed a detectable overall PFE difference (*p* = 0.047), all values remained above the required threshold (Figure 5; Table S3) and effect magnitudes were marginal (Table S4).

### 3.7. Airflow Resistance

The airflow resistance (Δ*P*), a measure of breathability, of all tested SMs and FFRs was unaffected by a single dry-heat treatment cycle (Table 6). However, after multiple cycles, there were isolated and inconsistent changes, particularly among FFRs (Table 6 and Table S4).

Among SMs, the Level 2 ZA-S001B showed reduced breathability (higher airflow resistance) after two (median increase: +11.1 Pa; 95% CI: 4.4–14.0) and three cycles (+13.3 Pa; 95% CI: 9.9–15.7) compared to controls (Table 6 and Table S4). For RH-S919B (L2), Δ*P* decreased after three cycles (−8.6 Pa; 95% CI: −13.0 to −1.6; Table S4), despite a non-significant overall test (Table 6). Conversely, the Level 3 RH-S920TFG showed improved breathability (lower airflow resistance) after two (median decrease: −4.0 Pa; 95% CI: −8.9 to −3.2) and three cycles (−10.1 Pa; 95% CI: −15.0 to −6.2) (Table 6 and Table S4).

## 4. Discussion

### 4.1. Preservation of Protective Functions Despite Alterations in Mechanical Integrity

The key finding of this study is that the primary protective functions were preserved in all treated FFR replicates and in most treated SM replicates through three cycles of dry heat at 80 °C for 90 min, with failures confined to a single SM model. The functional performance remained despite multiple and complex alterations in the underlying mechanical properties of their constituent materials. Particle filtration efficiency of all treated FFR replicates (45/45) and most treated SM replicates (24/27) met performance thresholds [49,56], except for three treated RH-S919B replicates (two at 1× and one at 3×). Notably, these failures showed no cycle-related pattern (there were no PFE failures after two cycles), which is compatible with stochastic sample variability or lot-to-lot heterogeneity rather than progressive thermal damage under this protocol. Although we did not perform standard-compliant fluid-barrier testing of SMs, an exploratory synthetic blood splash challenge (Appendix A) is presented for transparency. These tests suggested no obvious changes in all but one replicate, but they should not be used to infer post-treatment regulatory compliance.

This apparent paradox (i.e., functional stability despite mechanical degradation) can be explained by the filtration mechanisms and the specific nature of the thermal stress applied. FFR filtration relies on the mechanical interception by the dense web of non-woven polypropylene fibres combined with the electrostatic attraction, where electret fibres carry quasi-permanent charges that electrostatically attract and capture aerosol particles [59]. The 80 °C treatment temperature remains well below the melting temperatures of polypropylene (*T_m_* ≈ 165–170 °C) and polyester (*T_m_* ≈ 250–260 °C) [60]. Therefore, our dry-heat treatment protocol did not cause melting or destruction of the fibre web architecture essential for effective mechanical filtration. The observed changes in mechanical properties, such as softening or stiffening, likely reflect more subtle alterations at the nanoscale and microscale structural levels, including changes in crystallinity, changes in free volume, relaxation of polymer chains, or annealing effects, rather than catastrophic structural failure at the macroscale level [61].

Importantly, the preservation of high PFE across all treated FFR replicates (45/45 meeting NIOSH N95 criteria) suggests that any loss of electrostatic charge under our dry-heat treatment protocol was unlikely to be functionally significant at the time of testing. This is a critical advantage over other disinfection methods, such as alcohol or soap and water, which neutralise the filter’s charge and severely compromise its efficiency [24,27,31]. Our observations are consistent with previous studies indicating that dry heat is among the least damaging methods for the electrostatic properties of FFRs [27,62]. However, we did not directly measure electrostatic charge or charge decay, and relative humidity inside the oven was not monitored, so smaller or transient humidity-related effects cannot be excluded. Future work should therefore combine similar dry-heat protocols with in situ RH logging and explicit electrostatic measurements. Still, our protocol at 80 °C for 90 min appears to operate within a temperature range hot enough to achieve broad-spectrum pathogen inactivation, but not so hot as to cause failure of the primary filtration or fluid barrier mechanisms. The mechanical degradation observed, while measurable, remains sub-critical to the essential protective functions of the PPE.

### 4.2. Material-Specific and Component-Specific Responses to Thermal Stress

Our findings showed that different PPE materials display highly heterogeneous responses to thermal stress. Their contrasting responses are likely a result of their specific polymer composition, the manufacturing process, and the component type, underscoring the fallacy of a “one-size-fits-all” assumption for PPE disinfection. This heterogeneity was evident across different models and even within a single item. The most striking example of a material-specific response was seen in the FFR fabrics. The Dräger DR-X1720C, made of polypropylene, became progressively stiffer with each heat cycle, whereas all tested 3M models, composed of polypropylene or polypropylene/polyester blends, became softer. Two models (3M-8210 and 3M-1860S) explicitly include polyester shell layers, and among the tested FFRs, 3M-8210 exhibited the most softening. This difference can be attributed to the polymers’ thermal properties, particularly the glass transition temperature (*T_g_*), the point at which an amorphous polymer transitions from a rigid, glassy state to a pliable, rubbery state [60,63].

The *T_g_* of polyethylene terephthalate (PET; a common polyester) is typically 70–80 °C, with amorphous PET often ≈75 °C [60]. Our 80 °C treatment protocol exceeds the *T_g_* of PET. Softening at *T_g_* is thermorheologically reversible (modulus recovers on cooling) unless the time above *T_g_* permits structural or morphological changes (e.g., relaxation of molecular orientation or changes in crystallinity) [64]. Because our tensile tests were performed near room temperature (below *T_g_* for PET), yet *E* remained lower after cooling, the reductions are more consistent with morphological/structural changes accrued during the above-*T_g_* dwell than with a purely reversible glass–rubber transition (structural relaxation in PET glassy state after above-*T_g_* annealing) [65]. By contrast, most SM layers are polypropylene (PP), whose *T_g_* is well below room temperature (≈ −10 °C) and *T_m_* ≈ 160–165 °C; therefore, *T_g_*-based arguments do not apply to PP at 80 °C [66]. Any induced changes at 80 °C in PP-based non-woven fabrics more likely reflect viscoelastic stress relaxation of oriented filaments or web-bonding effects rather than a glass transition [67]. For context, Table 7 summarises typical glass transition, melting, and thermal degradation temperatures for polypropylene, polyethylene terephthalate, and polyester-based thermoplastic elastomers used in the PPE models tested.

Furthermore, the change in mechanical properties (softening, rather than chemical degradation of the polymer) was sometimes non-monotonic, with stiffness appearing to partially recover or even increase after additional heat cycles, which suggests competing thermally induced processes. The first cycle may relax internal processing stresses, leading to softening; a subsequent cycle may permit annealing/recrystallisation, increasing stiffness and strength (PET annealing/relaxation and recrystallisation near/above *T_g_*) [72]. Finally, cumulative processes can yield non-monotonic trends: early stress-relaxation may be followed by annealing/recrystallisation that increases stiffness; true chemical chain scission (e.g., PET hydrolysis) typically requires moisture and longer durations than our short, dry 80 °C cycles and is therefore unlikely under the present conditions [73,74]. For RH-S919B in particular, the combination of lower stiffness and greater extensibility suggests a softer, more deformable fabric that could, in principle, alter mask shape during wear. However, we did not measure fit in this study, so any impact on performance during use remains uncertain.

### 4.3. Strap Integrity and the Critical Implications for Respirator Fit

While the filter media and fluid barriers performed well, the data on FFR strap mechanical degradation raise important questions about a critical aspect of respirator function not directly measured in this study: the facial seal or “fit.” An FFR is only effective if it forms a tight seal against the wearer’s face. Any gaps create a path of lower resistance, allowing contaminated air to be inhaled and bypass the filter entirely [75]. The integrity of the elastomeric straps is paramount for creating and maintaining this seal. In practice however, minor upper-strap displacement may not necessarily compromise fit, as shown in a pilot study, where 33 out of 35 FFRs still passed fit testing after the upper strap was moved down to the ear-sulcus level [76].

Our results show that the straps of several FFR models underwent substantial mechanical changes. The thermoplastic elastomer straps of the 3M-8210 and 3M-9320A+ models were weakened (decreased *σ_UTS_*) and, in the case of the 3M-8210, much stretchier (increased *ε_f_*) after repeated treatments. This combination of reduced strength and increased ductility suggests a loss of elastic recovery. For the 3M-8210 strap specifically, yield strength defined using the 0.2% strain-offset method fell from 0.43 to 0.08 MPa after a single cycle and remained substantially below baseline after two and three cycles (Table 5), implying that permanent set would occur at much lower strap tensions than in untreated devices. In practical terms, this makes it easier for the strap to plastically elongate under normal donning forces, reducing its ability to maintain tension over time. However, for elastomeric straps, the 0.2% offset yield point marks the stress at which the stress–strain curve deviates from linearity; it does not correspond to a specific damage threshold. For this reason, *σ_y_* should be interpreted together with changes in modulus, *σ_UTS_*, and *ε_f_*, rather than on its own as a predictor of fit. Such straps may fail to provide the consistent tension required to hold the respirator securely against the face, or they may become permanently stretched (‘baggy’) after repeated donning and doffing, leading to fit failure.

The stress–strain curves in Figure 3 provide visual evidence of material-dependent differences in strap behaviour. The braided polyisoprene straps of the 3M-1860S maintained very similar stress–strain profiles across all treatment levels, with essentially unchanged stiffness, strength, and ductility. This pattern is consistent with a chemically crosslinked elastomeric network that undergoes little thermal reorganisation under the relatively mild conditions used (80 °C). In contrast, the thermoplastic elastomer straps of the 3M-8210 showed a marked change in mechanical response after repeated dry-heat cycles: treated samples exhibited lower ultimate tensile strength (approximately 12–18 MPa versus 19–21 MPa in controls) and substantially greater strain at failure (up to 1400–1800% versus 800–1000% in controls). This combination of reduced strength and increased ductility is characteristic of thermally induced softening of thermoplastic elastomers, likely reflecting relaxation of molecular orientation or microstructural changes. From a functional perspective, these alterations suggest that the straps are likely to stretch more easily under normal donning forces, to accumulate permanent set more readily, and to provide less stable tensile loading over time—factors that may compromise maintenance of an adequate facial seal during respirator use.

Consistent with prior work on FFR straps, thermoplastic straps can exhibit viscoelastic relaxation and set with use, whereas braided polyisoprene (thermoset) straps tend to be comparatively stable; however, storage-age effects are model-dependent and can still occur [77,78]. Taken together, our data therefore indicate that thermoplastic elastomer straps are more vulnerable to loss of strength and elastic recovery after repeated dry-heat cycles than braided polyisoprene straps, and that this material-dependent divergence provides a mechanistic basis for the model-specific risks to fit and durability that we emphasise in this study. Empirical data show that repeated donning/doffing degrades strap tension and reduces protection factors: in a controlled study, the FFR protection factor declined substantially by the eighth donning/doffing cycle, requiring strap-length adjustment to preserve protection [77]. This aligns with stockpile data indicating that strap tensile properties can shift with storage age in some models (though effects may vary by manufacturer and material) [78].

Although this study did not include quantitative fit testing, fit is a common failure point for reused respirators, often due to handling and strap degradation [75]. Conversely, the preservation of ultimate tensile strength in the straps of the 3M-1860S, 3M-1870+, and Dräger models was a positive indicator for their potential reusability. In fact, these two 3M FFRs passed quantitative fit testing after four sequential cycles of dry-heat treatment at 80 °C for 60 min [42].

Nonetheless, our variable findings on strap mechanics provide a strong mechanistic basis for the fit test failures reported elsewhere. Further, they emphasise that future studies on PPE disinfection must include direct, quantitative fit testing as a primary safety endpoint. This is important as the likelihood of adverse effects of heat treatment on FFR fit varies widely [27]. A recent systematic review of 36 studies likewise found that fit-factor outcomes after dry-heat reprocessing are model- and method-dependent [79]. These observations are unsurprising given the heterogeneous material compositions and varying disinfection protocols across studies.

### 4.4. Implications for Public Health, Sustainability, and Pandemic Preparedness

These findings have several important implications for pandemic preparedness and the sustainability of PPE. The COVID-19 pandemic exposed major vulnerabilities in the global PPE supply chain [1,2], demonstrating that reliance on a linear, single-use consumption model is not resilient during a global health crisis. Given the high probability of future novel pandemics [21], establishing strategies for the safe reuse of PPE is an essential component of effective preparedness. Our specific, accessible, and low-cost dry-heat reprocessing method could be implemented relatively easily in multiple settings, ranging from large hospitals to smaller clinics and laboratories without access to specialised equipment, such as vaporised hydrogen peroxide (VHP) or large-scale UVGI systems.

Our national survey of more than 1400 healthcare workers in NZ found that PPE was commonly reused during the COVID-19 pandemic [80]. Most importantly, 85% of respondents reported being comfortable wearing disinfected PPE under certain circumstances (e.g., if there was evidence that the practice was safe). Two out of three (64%) respondents were comfortable doing so during supply shortages, whereas 15% were not comfortable reusing disinfected PPE under any circumstances [80]. Disinfection-and-reuse pathways could also mitigate the environmental impact of PPE waste observed during the pandemic [14]; notably, 76% of the NZ survey respondents rated reducing medical waste from single-use products as “somewhat important” or “very important” [80].

Our findings support the potential safe reuse of FFRs and SMs after up to three treatment cycles, contingent on model-specific function and fit testing for regulatory compliance, offering a practical pathway toward a more circular economy for PPE. This dry-heat reprocessing system could markedly reduce, at a local level at least, the consumption of plastic polymers used in PPE, while diverting vast quantities of plastic waste from landfills and reducing the PPE contamination that threatens aquatic and terrestrial ecosystems. Therefore, a hybrid strategy that combines stockpiling of single-use items with validated protocols and infrastructure for disinfection and reuse could potentially create a healthcare system that is more resilient, cost-effective, and environmentally sustainable.

However, published work indicates degradation of straps after repeated donning/doffing cycles (≈8 cycles in one study) [77] and storage-age effects on strap tension in some stockpiled models [78]. Consequently, when considering limited reuse under dry-heat protocols, it would be essential to implement (i) pre- and post-use user seal checks and periodic quantitative fit testing [55], (ii) visual/tactile inspection for strap elongation or ‘bagging’, and (iii) conservative cycle caps unless ongoing fit testing verifies seal retention.

### 4.5. Limitations and Future Research

The principal limitation of this study is the absence of post-treatment quantitative fit testing for FFRs. Our mechanical analysis of the straps indicates that our dry-heat treatment protocol may reduce fit factors in some FFR models. Future studies must integrate quantitative fit testing as a primary endpoint to assess the potential impact of dry-heat treatment on FFR facial seal over multiple cycles, in line with the mandatory regulatory requirements for tight-fitting respirators [55]. The heterogeneity summarised in a recent systematic review of 36 studies underscores why fit testing must be both model- and method-specific [79], and why acceptable fit cannot be assumed *a priori* even when filtration remains intact.

Beyond heat, real-world donning/doffing introduces viscoelastic set and tension loss, which can degrade protection factors over time. One study observed a marked drop after ≈8 donning/doffing cycles without heat [77]. Also, the strap properties in stockpiled respirators may differ due to storage time and manufacturing variations [78], making periodic verification necessary. Consequently, future work should pair extended-wear simulations with heat cycles and embed fit testing [55] as a key outcome. In addition, the PFE failure of three SM replicates highlights the need to include larger sample sizes and to record both batch/lot and storage metadata to quantify within-model heterogeneity (including potential stockpile effects).

The functional tests in this study (PFE and airflow resistance) were performed on only three replicates per model and treatment level (*n* = 3), limiting the precision of our estimates and our ability to characterise within-model variability fully. In addition, we did not monitor or log relative humidity inside the oven during treatment, and transient local increases due to moisture desorption from materials cannot be excluded. However, because no water or humidification was added and the chamber was operated at 80 °C, psychrometric estimates based on typical indoor laboratory conditions (≈20–25 °C, 40–60% RH) suggest the overall RH at 80 °C would be ≈2–4%; i.e., conditions unlikely to be a meaningful source of bias in our study. Nonetheless, taken together, the small sample size and isolated SM PFE failures underscore the need for larger replicate numbers and detailed batch/lot metadata in future work.

We also did not directly characterise microstructural changes in the filter media (e.g., fibre morphology, pore size distribution, thickness, or basis weight) or measure electrostatic surface charge/charge decay. Consequently, our mechanistic interpretations regarding softening, stiffening, or potential compaction of the non-woven webs are indirect and should be confirmed through studies incorporating scanning electron microscopy, porosimetry (or equivalent pore-size analyses), thickness and basis-weight measurements, and electrostatic characterisation under controlled humidity.

Regarding design intent and expected heterogeneity, the respirators and surgical masks we evaluated were not designed or developed with dry-heat reprocessing as a performance objective. Accordingly, model-specific differences in response to off-label dry-heat reprocessing are expected, reflecting legitimate differences in materials, construction, and manufacturing tolerances. Our selection was driven by availability in NZ during the study period, and cross-model contrasts are descriptive under our experimental protocols, rather than a head-to-head brand comparison or a generalisable ranking.

An additional limitation is that we did not perform new microbial inactivation assays on these specific PPE batches under the same tested conditions. The choice of 80 °C for 90 min was based on our previous work performed at the PC3 lab, where SARS-CoV-2 was first isolated and propagated in NZ at the time [81]. We reported ≥7 log_10_ reduction in SARS-CoV-2 at 60–70 °C [37], with additional data showing ≥6 log_10_ reduction in heat-tolerant bacterial pathogens at 75 °C for 90 min [46]. Thus, the present study should be interpreted as a materials and performance complement to those microbiological data, rather than as an independent verification of bioburden reduction for each model at 80 °C, particularly given that model/material characteristics and soil load could influence inactivation efficacy.

Further, our testing protocol was limited to three dry-heat cycles with no ‘real-world’ use in between cycles. While this provides valuable data for limited reuse scenarios, the degradation mechanisms that may dominate after more cycles (e.g., 5 or 10) remain unknown. Practical experience suggests that combined extended wear and repeated heat treatment may accelerate degradation compared to heat treatment alone. The effects of extended use in the real world (including soiling from sweat, oils, or makeup, and repeated donning/doffing) on disinfection efficacy, strap performance, and material degradation need to be assessed in future investigations.

Lastly, a comprehensive life-cycle assessment and cost–benefit analysis would be invaluable to quantify the potential economic and environmental benefits of implementing this treatment and reuse strategy at different scales. These data would strengthen the evidence base for safe and sustainable PPE practices.

## 5. Conclusions

In this laboratory study, up to three sequential cycles of dry-heat treatment at 80 °C for 90 min did not measurably degrade particle-filtration efficiency or breathability in any treated FFR replicates and most of the treated SM replicates (24/27, with three failures in one SM model and no cycle-related pattern). These findings indicate the potential for limited reuse, under comparable conditions, of a range of FFRs and SMs commercially available during the COVID-19 pandemic, despite inducing measurable, non-linear, and material-specific changes in the mechanical properties of the fabrics and straps. Under this protocol, all treated FFRs and most treated SMs retained their protective performance; however, the three PFE failures in one SM model and the observed mechanical changes warrant model-specific testing and quantitative fit verification. In particular, strap integrity merits routine checks (tension/elongation, visible ‘bagging’) during any reuse program, as repeated donning/doffing can measurably erode protection [77] and stockpiled straps can age heterogeneously [78]. Together with the absence of a cycle-related trend in the SM PFE failures, these considerations underscore the importance of larger replicate numbers to better characterise within-model variability.

The evidence suggests an apparent disconnect between immediate functional testing and material integrity. While standard performance metrics (filtration and breathability) remained largely intact, the substantial mechanical changes observed (including softening and, in some cases, reduced ductility consistent with embrittlement) suggest potential compromises in structural resilience. This could manifest as premature failure during donning or doffing, an inability to maintain a proper facial seal over time, or reduced protection under mechanical stress. Therefore, additional ‘real-world’ testing on currently available PPE is essential to evaluate the effects of alternating wear–treatment cycles (FFR/SM use interleaved with dry-heat treatment) under controlled conditions (i.e., without exposing wearers to infection risk). If their function and usability meet the required safety standards, this would validate dry-heat treatment as an efficient, accessible, and scalable method for reprocessing and potential reuse, contingent on model-specific fit validation. Such validation would provide robust evidence to support PPE supply chain resilience, reduce the environmental burden of medical waste, and strengthen global preparedness for future public health crises.

## Figures and Tables

**Table 1:** The types, models, manufacturers, and main material composition of tested surgical masks (SMs) and filtering facepiece respirators (FFRs) used in New Zealand by the Ministry of Health during the COVID-19 pandemic.

PPE Type	Model	Manufacturer (Country)	Fabric Composition	Strap Composition
Surgical masks	RH-S919B (L2)	Reynard (Wuhan, China)	Polypropylene	n/a
	ZA-S001B (L2)	Zuru (Qingyuan, China)	Polypropylene	n/a
	RH-S920TFG (L3)	Reynard (Wuhan, China)	Polypropylene	n/a
FFRs	DR-X1720C	Dräger (Lübeck, Germany)	Polypropylene	Woven elastic textile (latex-free)
	3M-1860S	3M (Saint Paul, MN, USA)	Polypropylene (filter), polyester (shell)	Braided polyisoprene
	3M-1870+	3M (Saint Paul, MN, USA)	Polypropylene	Braided polyisoprene
	3M-8210	3M (Hwaseong, South Korea)	Polypropylene (filter), polyester (shell, coverweb)	Thermoplastic elastomer
	3M-9320A+	3M (Singapore)	Polypropylene	Thermoplastic elastomer

For surgical masks, Levels 2 (L2) and 3 (L3) refer to their ASTM F2100-19 performance classification [49]. “n/a” indicates that the information was not collected, as these were not tested. The FFR models are NIOSH-certified N95 respirators (3M 1860S, 3M 1870+, 3M 8210) or P2/FFP2 class particulate respirators (Dräger DR X1720C, 3M 9320A+) that were widely used as N95-equivalent respiratory personal protective equipment (PPE) in NZ during the COVID-19 pandemic; these are referred to collectively as FFRs in this study. Model names are provided solely for accurate reporting and reproducibility; no comparative ranking, endorsement, or disparagement is intended. Gray shading distinguishes the information for the different PPE types.

**Table 2:** The meaning of changes in each tensile metric.

Parameter	Decrease	Increase
Young’s modulus (*E*)	Material becomes softer/more compliant	Material becomes stiffer/more rigid
	• Deforms more easily under the same load	• Greater resistance to deformation
	• More flexible	• Less flexible
Yield strength (*σ_y_* )	Lower resistance to permanent deformation	Higher resistance to permanent deformation
	• Starts deforming permanently at lower stress	• Can withstand greater stress before yielding
	• Weaker elastic region	• Stronger elastic region
Ultimate tensile strength (*σ_UTS_*)	Material becomes weaker	Material becomes stronger
	• Fails at lower loads	• Can bear higher loads before tensile failure
	• Reduced load capacity	• Greater load capacity
Strain at failure (*ε_f_*)	Material becomes more brittle	Material becomes more ductile
	• Reaches the failure criterion with less elongation	• Can stretch more before reaching the failure criterion
	• Reduced stretching ability	• Greater elongation capacity

Definitions of *E*, *σ_y_*, *σ_UTS_*, and *ε_f_* adapted from ISO 527-1 [53]. Gray shading distinguishes the information for the different parameters.

**Table 3:** Effects of multiple dry-heat treatment cycles on the mechanical properties of surgical mask fabrics.

Level	Model	Parameter	Control	1× Heat T_x_	2× Heat T_x_	3× Heat T_x_	*p*
**L2**	**RH-S919B**	*E* (MPa)	56.0 ± 6.8	31.9 ± 2.7 ****	38.6 ± 0.3 ****	24.9 ± 1.2 ****	**<0.0001**
		*σ_y_* (MPa)	2.36 ± 0.13	2.66 ± 0.23	3.14 ± 0.38 **	3.87 ± 0.42 ****	**<0.0001**
		*σ_UTS_* (MPa)	3.44 ± 0.17	2.84 ± 0.27 **	5.17 ± 0.33 ****	4.94 ± 0.35 ****	**<0.0001**
		*ε_f_* (%)	14.8 ± 2.5	10.7 ± 1.5	36.2 ± 9.8 ***	34.2 ± 12.3 **	**0.0001**
**L2**	**ZA-S001B**	*E* (MPa)	30.3 ± 1.2	20.3 ± 0.6 ****	17.9 ± 0.5 ****	30.4 ± 0.8	**<0.0001**
		*σ_y_* (MPa)	2.14 ± 0.19	2.17 ± 0.09	1.93 ± 0.11 *	1.30 ± 0.04 ****	**<0.0001**
		*σ_UTS_* (MPa)	3.42 ± 0.48	2.54 ± 0.20 ***	2.72 ± 0.23 **	2.31 ± 0.27 ****	**0.0003**
		*ε_f_* (%)	28.5 ± 8.9	17.1 ± 3.2 **	25.9 ± 3.4	18.3 ± 5.2 *	**0.013**
**L3**	**RH-S920TFG**	*E* (MPa)	57.4 ± 3.7	29.1 ± 0.9 ****	28.5 ± 1.8 ****	28.3 ± 2.0 ****	**<0.0001**
		*σ_y_* (MPa)	1.73 ± 0.29	2.62 ± 0.07 ****	2.34 ± 0.09 ****	2.34 ± 0.11 ****	**<0.0001**
		*σ_UTS_* (MPa)	4.41 ± 0.38	3.65 ± 0.20 ***	3.65 ± 0.27 ***	3.02 ± 0.15 ****	**<0.0001**
		*ε_f_* (%)	40.5 ± 4.9	21.5 ± 2.5 ****	27.6 ± 6.8 ***	19.1 ± 4.6 ****	**<0.0001**

*E*, Young’s modulus; *ε_f_*, strain at failure; *σ_UTS_*, ultimate tensile strength; *σ_y_*, yield strength. Each dry-heat treatment cycle consisted of 90 min at 80 °C in a fan-forced oven; 1×, 2×, and 3× Heat T_x_ indicate one, two, or three sequential cycles under these same conditions. Data are reported as mean ± standard deviation. The *p*-value for an overall difference between treatment levels was obtained using analysis of variance, and is highlighted in bold if statistically significant at *p* < 0.05; planned pairwise comparisons between treated and control samples were assessed using Fisher’s Least Significant Difference tests, where * *p* < 0.05, ** *p* < 0.01, *** *p* < 0.001, and **** *p* < 0.0001. Levels 2 (L2) and 3 (L3) refer to their ASTM F2100-19 performance classification [49]. Model names are provided solely for accurate reporting and reproducibility; no comparative ranking, endorsement, or disparagement is intended. Gray shading distinguishes data for the different models.

**Table 4:** Effects of multiple dry-heat treatment cycles on the mechanical properties of fabric from filtering facepiece respirators.

Model	Parameter	Control	1× Heat T_x_	2× Heat T_x_	3× Heat T_x_	*p*
**DR-X1720C**	***E*** ** (MPa)**	5.19 ± 0.36	9.52 ± 1.03 ****	8.79 ± 1.81 ***	11.42 ± 1.22 ****	**<0.0001**
	***σ_y_*** ** (MPa)**	1.33 ± 0.08	0.68 ± 0.18 **	1.82 ± 0.44 **	1.48 ± 0.15	**<0.0001**
	***σ**_UTS_* **(MPa)**	1.62 ± 0.13	1.18 ± 0.14 *	2.31 ± 0.49 **	2.08 ± 0.19 *	**<0.0001**
	***ε_f_*** ** (%)**	46.0 ± 4.3	31.3 ± 6.8 ***	38.8 ± 7.4 **^†^**	31.1 ± 2.6 ***	**0.002**
**3M-1860S**	***E*** ** (MPa)**	57.1 ± 11.9	63.7 ± 4.8	22.8 ± 4.8 ****	26.2 ± 4.8 ****	**<0.0001**
	***σ_y_*** ** (MPa)**	1.62 ± 0.52	2.02 ± 0.33	0.83 ± 0.57 *	0.72 ± 0.33 **	**0.003**
	***σ**_UTS_* **(MPa)**	2.36 ± 0.69	2.91 ± 0.62	2.17 ± 0.32	2.10 ± 0.53	0.24
	***ε_f_*** ** (%)**	12.2 ± 2.8	12.1 ± 8.6	13.7 ± 6.9	16.5 ± 3.6	0.59
**3M-1870+**	***E*** ** (MPa)**	40.9 ± 4.6	37.8 ± 5.3	29.2 ± 2.0 ***	36.2 ± 1.8 **^†^**	**0.001**
	***σ_y_*** ** (MPa)**	3.49 ± 0.24	2.72 ± 0.20 ***	3.54 ± 0.28	3.65 ± 0.27	**<0.0001**
	***σ**_UTS_* **(MPa)**	4.29 ± 0.28	3.38 ± 0.40 **	3.80 ± 0.38 ^†^	3.94 ± 0.49	**0.016**
	***ε_f_*** ** (%)**	25.0 ± 4.8	20.4 ± 4.7	19.6 ± 7.0	14.7 ± 5.3 **	**0.067**
**3M-8210**	***E*** ** (MPa)**	64.0 ± 12.4	11.6 ± 5.9 ****	27.2 ± 7.1 ****	17.7 ± 4.1 ****	**<0.0001**
	***σ_y_*** ** (MPa)**	1.67 ± 0.24	0.02 ± 0.01 ****	0.78 ± 0.25 ****	1.20 ± 0.18 **	**<0.0001**
	***σ**_UTS_* **(MPa)**	2.83 ± 0.55	2.56 ± 0.16	1.62 ± 0.29 ***	1.92 ± 0.42 **	**0.0004**
	***ε_f_*** ** (%)**	17.0 ± 1.9	14.4 ± 4.1	17.8 ± 3.6	18.3 ± 2.5	0.25
**3M-9320A+**	***E*** ** (MPa)**	31.9 ± 2.0	55.2 ± 4.9 ****	34.1 ± 2.1	26.5 ± 4.7 *	**<0.0001**
	***σ_y_*** ** (MPa)**	3.52 ± 0.28	2.70 ± 0.43 **	3.22 ± 0.10	3.49 ± 0.47	**0.007**
	***σ**_UTS_* **(MPa)**	3.78 ± 0.35	3.40 ± 0.77	3.48 ± 0.25	3.68 ± 0.53	0.65
	***ε_f_*** ** (%)**	18.7 ± 5.3	15.7 ± 6.8	16.5 ± 3.9	20.1 ± 8.5	0.68

*E*, Young’s modulus; *ε_f_*, strain at failure; *σ_UTS_*, ultimate tensile strength; *σ_y_*, yield strength. Each dry-heat treatment cycle consisted of 90 min at 80 °C in a fan-forced oven; 1×, 2×, and 3× Heat T_x_ indicate one, two, or three sequential cycles under these same conditions. Data are reported as mean ± standard deviation. The *p*-value for an overall difference between treatment levels was obtained using analysis of variance, and is highlighted in bold if statistically significant at *p* < 0.05; planned pairwise comparisons between treated and control samples were assessed using Fisher’s Least Significant Difference tests, where ^†^ *p* ≤ 0.07, * *p* < 0.05, ** *p* < 0.01, *** *p* < 0.001, and **** *p* < 0.0001. Model names are provided solely for accurate reporting and reproducibility; no comparative ranking, endorsement, or disparagement is intended. Gray shading distinguishes data for the different models.

**Table 5:** Effects of multiple dry-heat treatment cycles on the mechanical properties of straps from filtering facepiece respirators.

Model	Parameter	Control	1× Heat T_x_	2× Heat T_x_	3× Heat T_x_	*p*
**DR-X1720C**	***E*** ** (MPa)**	0.37 ± 0.05	0.48 ± 0.08	0.61 ± 0.20 **	0.51 ± 0.14	0.064
	***σ_y_*** ** (MPa)**	0.020 ± 0.004	0.021 ± 0.002	0.027 ± 0.002 **	0.025 ± 0.005 *	**0.013**
	***σ**_UTS_* **(MPa)**	14.5 ± 0.9	14.9 ± 1.1	15.5 ± 0.8	15.3 ± 0.4	0.26
	***ε_f_*** ** (%)**	233 ± 42	175 ± 31 *	184 ± 38 ^†^	212 ± 38	0.09
**3M-1860S**	***E*** ** (MPa)**	0.70 ± 0.08	0.71 ± 0.04	0.59 ± 0.04 *	0.55 ± 0.08 **	**0.001**
	***σ_y_*** ** (MPa)**	0.043 ± 0.004	0.033 ± 0.005	0.061 ± 0.033	0.084 ± 0.011 **	**0.002**
	***σ**_UTS_* **(MPa)**	23.9 ± 1.2	22.4 ± 0.7	23.7 ± 1.0	23.9 ± 1.7	0.19
	***ε_f_*** ** (%)**	412 ± 48	332 ± 17 **	379 ± 54	424 ± 35	**0.012**
**3M-1870+**	***E*** ** (MPa)**	0.65 ± 0.03	0.47 ± 0.03 ****	0.55 ± 0.06 **	0.42 ± 0.07 ****	**<0.0001**
	***σ_y_*** ** (MPa)**	0.046 ± 0.016	0.055 ± 0.015	0.110 ± 0.033 ***	0.126 ± 0.013 ****	**<0.0001**
	***σ**_UTS_* **(MPa)**	11.5 ± 0.7	11.4 ± 0.5	10.9 ± 0.7	10.9 ± 0.5	0.29
	***ε_f_*** ** (%)**	605 ± 85	658 ± 35	533 ± 63	806 ± 180 **	**0.006**
**3M-8210**	***E*** ** (MPa)**	0.30 ± 0.02	0.25 ± 0.09	0.75 ± 0.15 ***	0.69 ± 0.22 **	**<0.0001**
	***σ_y_*** ** (MPa)**	0.43 ± 0.03	0.08 ± 0.03 ****	0.16 ± 0.08 ***	0.19 ± 0.13 ***	**<0.0001**
	***σ**_UTS_* **(MPa)**	21.8 ± 0.7	19.2 ± 1.3 *	17.3 ± 1.9 **	15.0 ± 2.1 ****	**0.0001**
	***ε_f_*** ** (%)**	914 ± 13	854 ± 62	1453 ± 313 **	1469 ± 300 **	**<0.001**
**3M-9320A+**	***E*** ** (MPa)**	0.66 ± 0.04	0.59 ± 0.02 *	0.27 ± 0.06 ****	0.40 ± 0.03 ****	**<0.0001**
	***σ_y_*** ** (MPa)**	0.063 ± 0.005	0.057 ± 0.006	0.124 ± 0.018 ****	0.143 ± 0.011 ****	**<0.0001**
	***σ**_UTS_* **(MPa)**	10.5 ± 0.4	10.4 ± 0.7	8.9 ± 0.3 ***	9.1 ± 0.7 **	**0.0003**
	***ε_f_*** ** (%)**	482 ± 82	481 ± 72	866 ± 72 ****	535 ± 29	**<0.0001**

*E*, Young’s modulus; *ε_f_*, strain at failure; *σ_UTS_*, ultimate tensile strength; *σ_y_*, yield strength. Each dry-heat treatment cycle consisted of 90 min at 80 °C in a fan-forced oven; 1×, 2×, and 3× Heat T_x_ indicate one, two, or three sequential cycles under these same conditions. Data are reported as means ± standard deviations. The *p*-value for an overall difference between treatment levels was obtained using analysis of variance, and is highlighted in bold if statistically significant at *p* < 0.05; planned pairwise comparisons between treated and control samples were assessed using Fisher’s Least Significant Difference tests, where ^†^ *p* ≤ 0.07, * *p* < 0.05, ** *p* < 0.01, *** *p* < 0.001, and **** *p* < 0.0001. Model names are provided solely for accurate reporting and reproducibility; no comparative ranking, endorsement, or disparagement is intended. Gray shading distinguishes data for the different models.

**Table 6:** Effects of multiple dry-heat treatment cycles on the airflow resistance (breathability) of surgical masks (SMs) and filtering facepiece respirators (FFRs) tested.

PPE Type	Model	Control	1× Heat T_x_	2× Heat T_x_	3× Heat T_x_	*p*
**SMs**	**RH-S919B (L2)**	34.5 [28.3, 38.8]	30.7 [29.7, 32.1]	23.8 [22.5, 31.4]	25.9 [25.8, 26.7]	0.11
	**ZA-S001B (L2)**	16.2 [13.9, 16.3]	14.9 [14.1, 16.3]	27.3 [20.7, 27.9]	29.5 [26.2, 29.6]	**0.035**
	**RH-S920TFG (L3)**	36.7 [36.6, 41.5]	40.3 [37.8, 41.2]	33.0 [32.6, 33.4]	28.4 [26.5, 30.4]	**0.024**
**FFRs**	**DR-X1720C**	53.3 [38.4, 59.2]	53.4 [50.4, 54.9]	49.7 [46.3, 50.3]	88.2 [57.0, 114]	0.072
	**3M-1860S**	42.0 [28.5, 42.9]	32.6 [29.5, 38.8]	43.9 [39.8, 46.9]	48.8 [48.4, 49.9]	**0.038**
	**3M-1870+**	39.3 [35.2, 39.4]	35.9 [35.9, 47.0]	37.5 [36.8, 38.6]	37.2 [30.8, 37.7]	0.83
	**3M-8210**	44.7 [43.3, 48.2]	43.8 [42.5, 44.6]	44.5 [43.5, 46.1]	43.0 [41.5, 46.2]	0.62
	**3M-9320A+**	30.0 [30.0, 30.2]	30.1 [30.0, 31.9]	27.5 [27.1, 27.6]	28.2 [27.2, 30.3]	0.13

Each dry-heat treatment cycle consisted of 90 min at 80 °C in a fan-forced oven; 1×, 2×, and 3× Heat T_x_ indicate one, two, or three sequential cycles under these same conditions. Levels 2 (L2) and 3 (L3) refer to their ASTM F2100-19 performance classification [49]. Airflow resistance data are reported as the median [minimum, maximum] from three replicates, and expressed in Pa units. The *p*-value for an overall difference between treatment levels was obtained using a non-parametric Kruskal–Wallis test, and highlighted in bold if statistically significant at *p* < 0.05. Pairwise effect magnitudes were quantified using Wilcoxon-derived Hodges–Lehmann location shift estimates with 95% confidence intervals, and reported in Table S4 if there was evidence of a treatment effect. Model names are provided solely for accurate reporting and reproducibility; no comparative ranking, endorsement, or disparagement is intended. Gray shading distinguishes data for the different types of personal protective equipment (PPE).

**Table 7:** Approximate thermal transition and degradation temperatures for the main polymers used in the tested surgical masks and filtering facepiece respirators.

Polymer Type	*T_g_*	*T_m_* (°C)	*T_d_* (°C)	Sources
Polypropylene (PP)	−20 to 0 °C	160–175 °C	≈300–350 °C	[60,66,68,69]
Polyethylene terephthalate (PET)	70–80 °C	245–260 °C	≈350–430 °C	[60,70]
Thermoplastic elastomer (polyester-based)	≈0–20 °C ^1^	≈190–220 °C ^2^	>330 °C	[71]

*T_d_*, degradation temperature; *T_g_*, glass transition temperature; and *T_m_*, Melting Temperature. ^1^ Soft segment. ^2^ Hard segment melting. Values represent typical ranges for commercial grades under standard differential scanning calorimetry (DSC) and thermogravimetric analysis (TGA) conditions and may vary with molecular weight, processing history, additives, and test protocols. Manufacturer and database names are provided solely for accurate reporting and reproducibility.

**Figure A1:**
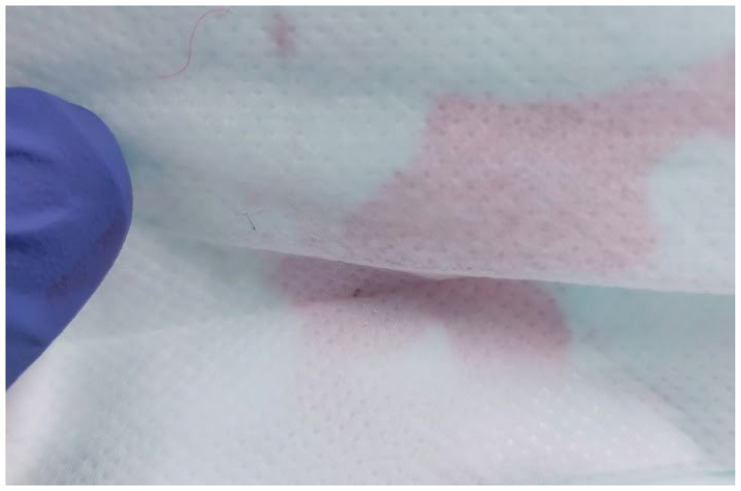
Evidence of an unsuccessful blood penetration test with ‘bloodstain’ detection on the inside surface of a Level 3 surgical mask following three cycles of dry-heat treatment.

## Data Availability

The original contributions presented in this study are included in the article/Supplementary Materials. Further inquiries can be directed to the corresponding author.

## Supplementary Materials

The following supporting information can be downloaded at: https://www.mdpi.com/article/10.3390/microorganisms14010069/s1, Figure S1: Mean temperature profiles of two 120 min oven cycles at 80 °C; Figure S2: Temperature at nine oven locations during two 120 min temperature-profiling runs; Table S1: Number of PPE replicates tested. Table S2: Direction of tensile parameter changes (observational summary); Table S3: Effects of dry-heat treatment cycles on PFE of SMs and FFRs; Table S4: Statistically significant pairwise comparisons between affected treated samples and controls; Dataset S1 (XLSX): Study dataset and accompanying data dictionary.

## Disclaimers

(1) *Scope of findings*—Results were derived from laboratory experiments and were not certification tests, clinical guidance, regulatory advice, or legal advice. Nothing herein modifies instructions for use (IFU), certifications, or approvals. (2) *Single-use & off-label context*—The evaluated PPE products were designed for single-use, and dry-heat reprocessing constitutes off-label use. Thus, post-treatment performance must not be assumed to meet manufacturer or regulatory specifications, and observations under off-label conditions do not indicate defects for the intended single-use purpose. (3) *Model identification*—Manufacturer and model names are provided solely for accurate reporting and reproducibility, and no comparative ranking, endorsement, or disparagement is intended. Our sample was availability-driven in New Zealand during the study period, and cross-model contrasts are descriptive, not generalisable brand comparisons. (4) *Independence*—This work was conducted independently without manufacturer sponsorship, input, or review. Any consideration of reuse requires local validation (including fit testing, where applicable) and adherence to institutional policy and regulation.

## Abbreviations

The following abbreviations are used in this manuscript:

*A*_0_	Initial cross-sectional area of specimen (width × thickness) during tensile testing
CI	Confidence intervals
*E*	Young’s modulus
*F*	Tensile load
*ε_f_*	Strain at failure
FDA	U.S. Food and Drug Administration
FFR	Filtering facepiece respirator
LMICs	Low- and middle-income countries
NIOSH	National Institute for Occupational Safety and Health
NZ	New Zealand
OSHA	Occupational Safety and Health Administration
PET	Polyethylene terephthalate
PFE	Particle filtration efficiency
PP	Polypropylene
PPE	Personal protective equipment
*σ_UTS_*	Ultimate tensile strength
*σ_y_*	Yield strength
SARS	severe acute respiratory syndrome
SARS-CoV-2	severe acute respiratory syndrome coronavirus 2
SM	Surgical mask
TCID_50_	50% tissue culture infectious dose
T_x_	Treatment
UVGI	Ultraviolet germicidal irradiation

## Appendix A. Exploratory Synthetic-Blood Splash Challenge of Surgical Masks

### Appendix A.1. Rationale

During drafting, we confirmed that the synthetic-blood formulation used for splash testing had a measured surface tension of 62.8 dyn·cm^−1^, whereas relevant mask splash standards (e.g., ASTM F1862/F1862M [50]) require a low-surface-tension fluid (≈40 dyn·cm^−1^). Because higher surface tension reduces wetting and penetration, the test is less stringent and therefore optimistically biased (i.e., it likely underestimates penetration under standard conditions). These data are presented solely for documentation purposes and must not be interpreted as evidence of fluid-barrier compliance.

### Appendix A.2. Methods

The splash challenge followed the sample geometry, mounting, distance, and challenge pressures specified in ASTM F1862/F1862M [50]. However, the test did not fully meet the standard because the synthetic-blood surface tension was higher than required. HPLC-grade water (0.975 L; pH 7.0 ± 0.5) (Sigma-Aldrich, 270733-4L, Burlington, MA, USA) was brought to a rolling boil for 5 min to minimise biological contamination and then allowed to air-cool to 20 ± 1 °C. Sodium polyacrylate (50 g; Acrysol 8306, Sigma-Aldrich, 447013-100G, Burlington, MA, USA) was added to the distilled water as a thickening agent and magnetically stirred until a homogeneous solution was obtained (≈45 min).

Direct Red 81 (10 g; Sigma-Aldrich, 195251-25G) was used as the colourant. A non-ionic surfactant was added, and the solution was magnetically stirred for one hour. The density of the synthetic blood was 1.032 g·cm^−3^, measured using a 10 mL density bottle and an analytical balance (Sartorius ED224S, Sartorius, Göttingen, Germany).

Surface tension was measured by the pendant-drop method with a KSV CAM200 goniometer (KSV Instruments Ltd., Monroe, CT, USA). Importantly, the surface tension of the synthetic blood was 62.8 dyn·cm^−1^, which is higher than the ≈40 dyn·cm^−1^ low-surface-tension requirement specified in ASTM F1862/F1862M standards for blood penetration testing of surgical masks [50].

Penetration testing was performed using a dedicated test apparatus (GT-RA01, Gester International Co., Ltd., Quanzhou, Fujian, China). Five replicates of each SM model were tested for each treatment level. Masks were conditioned at 21 ± 5 °C and 85 ± 5% relative humidity for at least 4 h, consistent with the environmental conditioning used for fluid-penetration tests (ASTM E171/E171M) [82]. Individual SMs were mounted onto a convex platform to retain their shape during testing, and a jet of synthetic blood was projected at their outer surface from a distance of 30.5 cm. Test pressures were set according to the mask’s designated ASTM level: 120 mmHg (16.0 kPa) for Level 2 masks and 160 mmHg (21.3 kPa) for Level 3 masks [50]. After a 10 s exposure, the inner surface of each mask was visually inspected using a magnifying glass for any evidence of fluid penetration. A pass was recorded if no penetration was observed.

### Appendix A.3. Results

Under these non-compliant conditions, all Level 2 masks (RH-S919B and ZA-S001B) passed at all treatment levels (5/5 each) (Table A1). For the Level 3 mask (RH-S920TFG), all control, 1×, and 2× replicates passed, but 1 of 5 failed after three treatment cycles (4/5 passes) with visible inner-surface staining (Figure A1).

**Table A1:** Pass/fail counts for blood penetration tests by the surgical mask model following repeated dry-heat treatment cycles.

**Model**	**ASTM Level**	**Test Pressure**	**Control**	**1× Heat T_x_**	**2× Heat T_x_**	**3× Heat T_x_**
RH-S919B	Level 2	120 mmHg	5/5	5/5	5/5	5/5
ZA-S001B	Level 2	120 mmHg	5/5	5/5	5/5	5/5
RH-S920TFG	Level 3	160 mmHg	5/5	5/5	5/5	4/5

T_x_, treatment. Red text indicates the group where a failure was observed.

### Appendix A.4. Conclusions

Higher surface tension reduces wetting and penetration, making the test less stringent. Since surface tension was high (62.8 dyn·cm^−1^), these findings likely underestimate the likelihood of penetration relative to standard conditions of ≈40 dyn·cm^−1^.

Nonetheless, we report these data to document the original work and to demonstrate that no widespread changes appeared to occur due to dry-heat treatment under these non-standard conditions. Still, these findings must not be used to infer regulatory compliance or clinical safety. Future testing of heat-treated SMs should repeat splash assessments with surface tension controlled to ≈40 dyn·cm^−1^ and tests in full alignment with the required standard. Only then can more robust conclusions about the function and safety of heat-treated SMs be ascertained.
