# Supplementary material for: From Bench to Breath: Material Integrity and Performance of Filtering Facepiece Respirators and Surgical Masks After Multi-Cycle Dry-Heat Reprocessing
Source: Microorganisms. 2025 Dec 29;14(1):69. doi: 10.3390/microorganisms14010069 (PMC12843907; doi:10.3390/microorganisms14010069)
Supplement: Supplementary file 1 [file microorganisms-14-00069-s001.zip › microorganisms-4002743-supplementary final.pdf]

SUPPLEMENTARY FILE

From Bench to Breath: Material Integrity and Performance of Filtering Facepiece Respirators and Surgical Masks After Multi-Cycle Dry-Heat Reprocessing

Mohammad Sagor Hosen[1,†], José G. B. Derraik[2,3†,*], Mohammad Shahbaz[4], William A. Anderson[5], Yvonne C. Anderson[2,6,7,8,‡], Mark P. Staiger[1,‡]

[1] Department of Mechanical Engineering, University of Canterbury, Christchurch, New Zealand.

[2] Department of Paediatrics: Child and Youth Health, Faculty of Medical and Health Sciences, University of Auckland, Auckland, New Zealand.

[3] Environmental–Occupational Health Sciences and Non-Communicable Diseases Research Centre, Research Institute for Health Sciences, Chiang Mai University, Chiang Mai, Thailand.

[4] Liggins Institute, University of Auckland, Auckland, New Zealand.

[5] Department of Chemical Engineering, University of Waterloo, Waterloo, Canada.

[6] Curtin Medical School, Faculty of Health Sciences, Curtin University, Bentley, Perth, Australia.

[7] The Kids Research Institute Australia, Perth, Australia.

[8] Child and Adolescent Community Health, Child and Adolescent Health Service, Perth, Australia.

† These authors share first authorship.

‡ These authors share last authorship.

***** Author for correspondence: j.derraik@auckland.ac.nz

FIGURE S1

Mean temperature profiles of two 120-minute oven cycles at 80 °C.
Black and blue curves represent the mean temperature across nine Type-T thermocouples distributed throughout the oven, sampled at 2-second intervals during two independent runs. The red horizontal line indicates the 80 °C target.

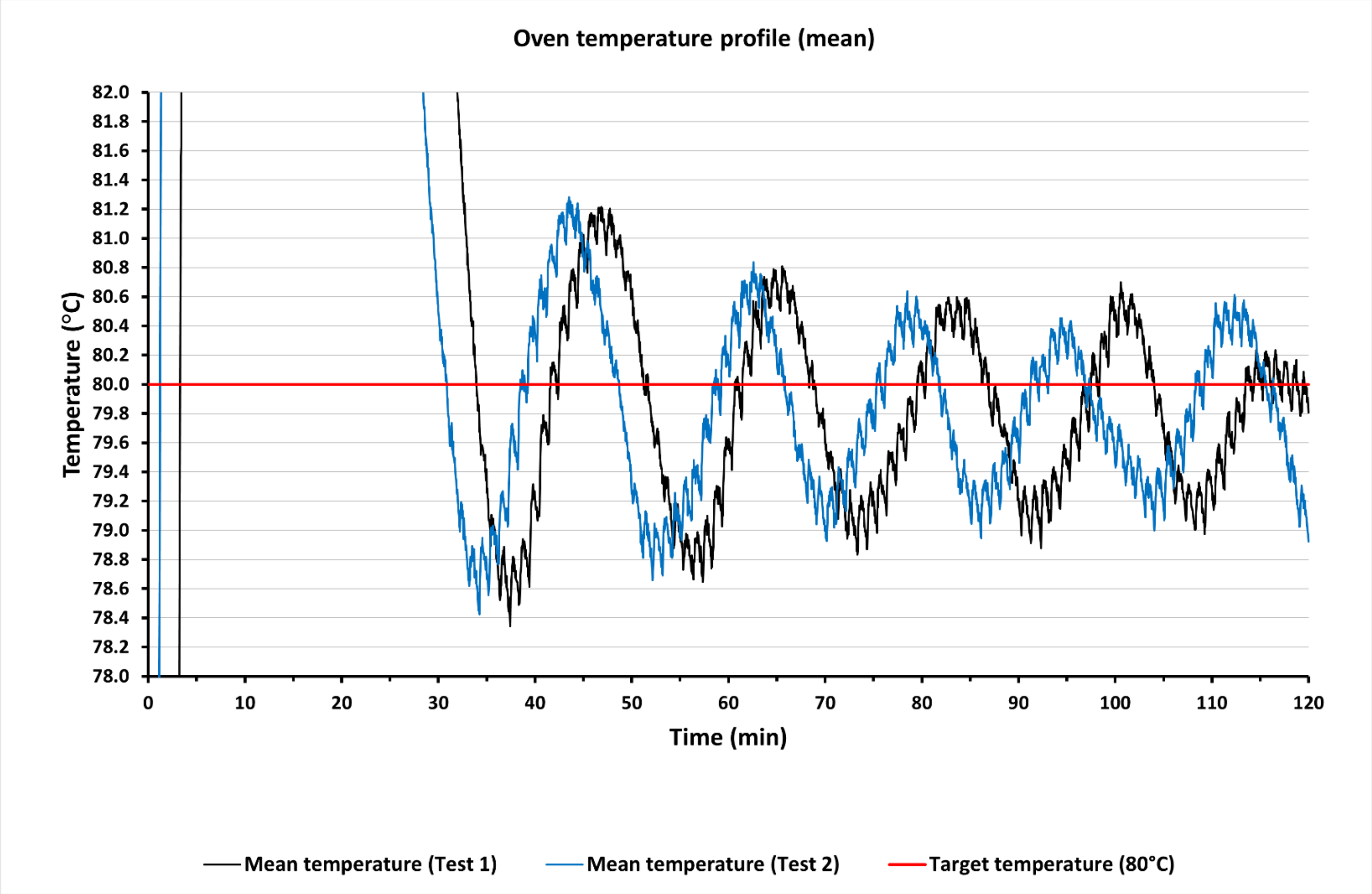

FIGURE S2

Temperature at nine oven locations during two 120-minute temperature-profiling runs.

Nine Type-T thermocouples positioned at top/bottom × front/back locations plus the centre recorded temperature at 2-second intervals throughout each 120-minute cycle.

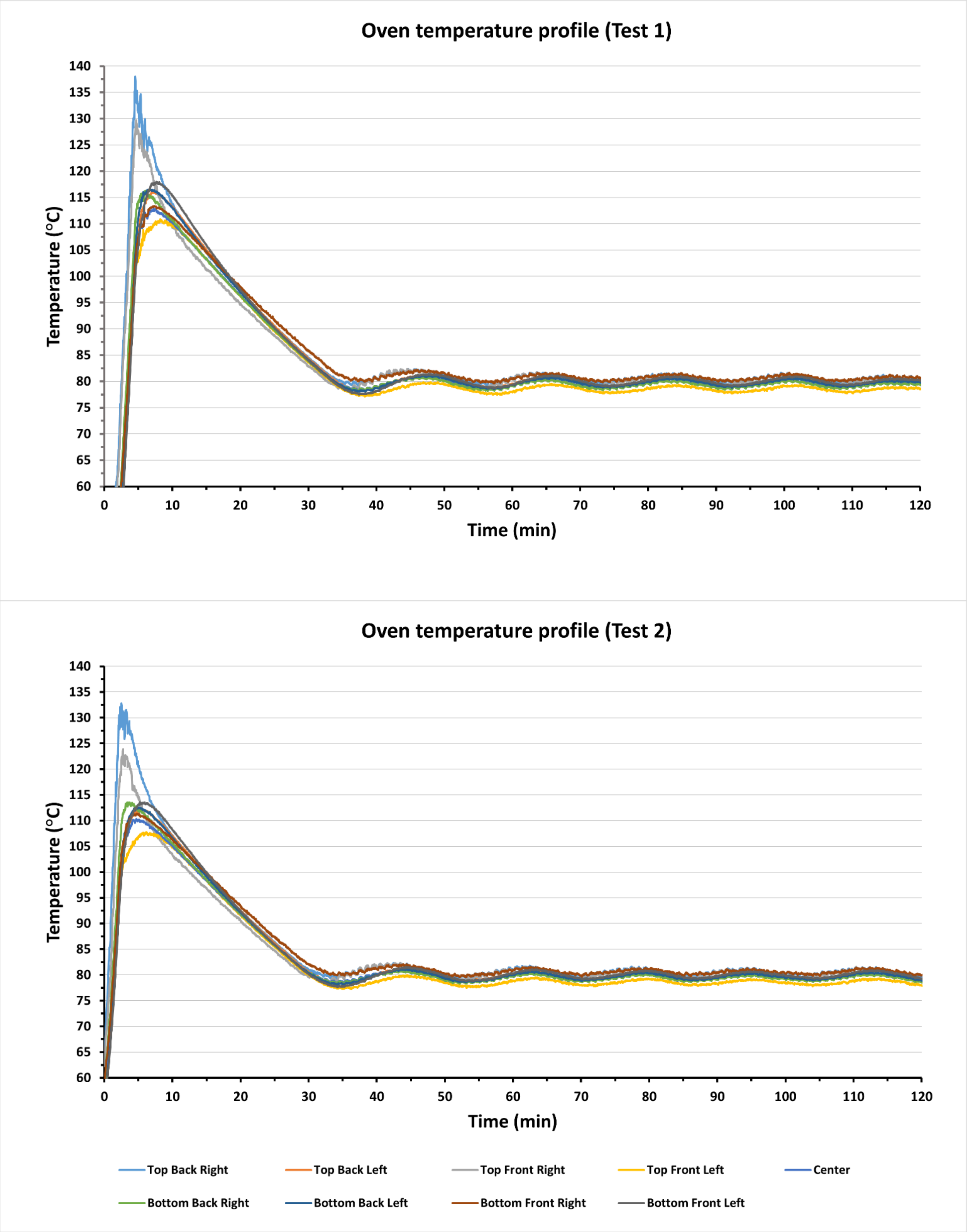

Table S1

Number of replicates used in this study according to test and PPE type.

All tests involved exposure of samples to four treatment (T_x) levels: control (untreated), and one, two, and three cycles of dry-heat treatment for 90 min at 80 °C in a fan-forced oven.

Tests	Parameters	Filtering facepiece respirators			Surgical masks		
		Models (*n*)	Replicates (*n*)	Total per T_x (*n*)	Models (*n*)	Replicates (*n*)	Total per T_x (*n*)
**Tensile properties**	*E*	5	5	25	3	5	15
	σ_y	5	5	25	3	5	15
	σ_{UTS}	5	5	25	3	5	15
	ε_f	5	5	25	3	5	15
	Subtotal per T_x			100			60
**Particle filtration efficiency**		5	3	15	3	3	9
	Subtotal per T_x			15			9
**Airflow resistance**		5	3	15	3	3	9
	Subtotal per T_x			15			9
**Total per T_x**				130			78
**Total per PPE type**				520			312

E, Young's modulus; ε_f, strain at failure; n/a, not applicable as not tested; PPE, personal protective equipment; σ_{UTS}, ultimate tensile strength; σ_y, yield strength; T_x, treatment.

TABLE S2

Direction of changes in tensile parameters by model and heat cycle (observational summary).

All tests involved exposure of samples to four treatment (T_x) levels: control (untreated), and one, two, or three cycles of dry-heat treatment for 90 min at 80 °C in a fan-forced oven. For each parameter tested, the effect of heat treatment was classified as "↑" (consistent increase); "↓" (consistent decrease); "~" (variable, non-linear, or conflicting changes); and "–" (no evidence of change).

		FFRs					Surgical masks		
**PPE part**	**Mechanical property**	**DR-X1720C**	**3M-1860S**	**3M-1870+**	**3M-8210**	**3M-9320A+**	**ZA-S001B (L2)**	**RH-S919B (L2)**	**RH-S920TFG (L3)**
**Fabric**	**Young's modulus (*E*)**	↑	↓ ~	↓ ~	↓	~	↓ ~	↓	↓
	**Yield strength (σ_y)**	~	↓ ~	~	↓	~	↓	↑	↑
	**σ_{UTS}**	~	–	↓ ~	↓	–	↓	↓	↓
	**Strain at failure (ε_f)**	↓	–	↓ ~	–	–	↓ ~	↑	↓
**Straps**	**Young's modulus (*E*)**	~	↓	↓	↑ ~	↓	n/a	n/a	n/a
	**Yield strength (σ_y)**	↑	↑ ~	↑	↓	↑	n/a	n/a	n/a
	**σ_{UTS}**	–	–	–	↓	↓	n/a	n/a	n/a
	**Strain at failure (ε_f)**	↓ ~	~	~	↑	~	n/a	n/a	n/a

FFRs, filtering facepiece respirators; L2, protection level 2; L3, protection level 3; PPE, personal protective equipment; σ_{UTS}, ultimate tensile strength. L2 and L3 refer to ASTM F2100–19 performance classifications requiring particle filtration efficiency ≥98%. Arrows indicate observed changes; interpretation is provided in the Discussion in the main manuscript. No mechanistic inference is made here. Model names are provided solely for accurate reporting and reproducibility; no comparative ranking, endorsement, or disparagement is intended.

TABLE S3

Effects of multiple dry-heat treatment cycles on the particle filtration efficiency of surgical masks (SMs) and filtering facepiece respirators (FFRs).

PPE part	Model	Control	1× Heat T_x	2× Heat T_x	3× Heat T_x	*p*
**Surgical masks**	**RH-S919B (L2)**	99.1 [98.5, 99.5]	97.7 [94.9, 98.2]	99.3 [98.6, 99.6]	99.5 [97.3, 99.7]	0.19
	**ZA-S001B (L2)**	99.1 [98.3, 99.5]	98.7 [98.4, 99.1]	99.7 [99.6, 99.8]	99.6 [99.3, 99.7]	**0.047**
	**RH-S920TFG (L3)**	99.7 [99.2, 99.9]	99.5 [99.4, 99.7]	99.8 [99.8, 99.9]	99.5 [99.5, 99.7]	0.19
**FFRs**	**DR-X1720C**	99.9 [99.7, 99.9]	99.7 [99.4, 99.8]	99.5 [98.6, 100]	99.5 [99.0, 100]	0.79
	**3M-1860S**	99.7 [99.3, 99.7]	99.7 [99.0, 100]	100 [99.3, 100]	98.3 [98.0, 100]	0.62
	**3M-1870+**	100 [99.8, 100]	100 [99.9, 100]	100 [99.7, 100]	99.8 [98.9, 100]	0.70
	**3M-8210**	99.8 [99.8, 100]	99.7 [99.5, 100]	99.0 [98.9, 100]	100 [99.7, 100]	0.47
	**3M-9320A+**	99.9 [99.8, 100]	100 [99.9, 100]	100 [98.3, 100]	100 [99.8, 100]	0.94

Each dry-heat treatment cycle consisted of 90 min at 80 °C in a fan-forced oven; 1×, 2×, and 3× Heat T_x indicate one, two, or three sequential cycles under these same conditions. Levels 2 (L2) and 3 (L3) refer to ASTM F2100–19 performance classifications requiring particle filtration efficiency ≥98%. Data are reported as the median [minimum, maximum] from three replicates, and expressed as the percentage of particles filtered. The overall difference between treatment levels was assessed using a non-parametric Kruskal–Wallis test, and the *p*-value is highlighted in bold if statistically significant at $p<0.05$. Pairwise effect magnitudes were quantified using Wilcoxon-derived Hodges–Lehmann location-shift estimates with 95% confidence intervals, and reported in Table S4 if there was evidence of a treatment effect. Values ≥99.94% to <100% were rounded to 99.9% to indicate that PFE was imperfect. Model names are provided solely for accurate reporting and reproducibility; no comparative ranking, endorsement, or disparagements is intended.

TABLE S4

Statistically significant differences in PPE performance metrics from planned pairwise comparisons between heat-treated and control samples.

Data are reported as Wilcoxon/Hodges–Lehmann median differences (treated − control) with 95% confidence intervals. All tests involved exposure of samples to four treatment levels: control (C; untreated), and one, two, and three cycles of dry-heat treatment (HT) for 90 min at 80 °C in a fan-forced oven.

Test	PPE type	Model	Pairwise comparison	Hodges-Lehmann location shift estimate
**Particle filtration efficiency (%)**	**SM**	**ZA-S001B (L2)**	**2×HT vs C**	0.6 (0.1–1.5)
**Airflow resistance (Pa)**	**FFR**	**3M-1860S**	**3×HT vs C**	7.0 (5.5–21.4)
		**3M-9320A+**	**2×HT vs C**	−2.6 (−3.1 to −2.4)
	**SM**	**RH-S919B (L2)**	**3×HT vs C**	−8.6 (−13.0 to −1.6)
		**RH-S920TFG (L3)**	**2×HT vs C**	−4.0 (−8.9 to −3.2)
			**3×HT vs C**	−10.1 (−15.0 to −6.2)
		**ZA-S001B (L2)**	**2×HT vs C**	11.1 (4.4–14.0)
			**3×HT vs C**	13.3 (9.9–15.7)

FFR, filtering facepiece respirators; L2, protection level 2; L3, protection level 3; PPE, personal protective equipment; SM, surgical masks. Only comparisons with statistically significant overall Kruskal–Wallis tests or non-trivial effect sizes are shown; pairwise comparisons without such effects are not reported. Model names are provided solely for accurate reporting and reproducibility; no comparative ranking, endorsement, or disparagement is intended.
